# Stepwise design and synthesis of red-emitting, reversible, HOCl chemosensors for monitoring the progression of drug-induced liver injury

**DOI:** 10.1039/d6ra04366f

**Published:** 2026-07-28

**Authors:** Chiqiong Liu†, Can Zeng, Fengying He, Wen-An Kuang

**Affiliations:** a School of Medicine, Hunan Polytechnic of Environment and Biology Hengyang China Liuchiqiong@outlook.com; b Hunan Xiangjiang New District Center for Disease Control and Prevention Changsha China; c The First Affliated Hospital, Department of Thoracic and Cardiovascular, Hengyang Medical School, University of South China Hengyang China

## Abstract

Drug-induced liver injury (DILI) is characterized by direct hepatocellular damage and liver dysfunction caused by pharmaceutical agents. However, effective diagnostic methods are currently lacking due to the absence of suitable biomarkers. Hypochlorous acid/hypochlorite (HOCl/ClO^−^) serves as a prodromal factor in DILI and holds promise as a novel biomarker for liver injury. Fluorescence imaging has made the real-time *in vivo* detection of HOCl possible. To achieve HOCl detection, we progressively designed three molecules: PTZ, DPTZ, and DPSZ, which successively realized fluorescence imaging of HOCl, extended the emission wavelength, and achieved reversible imaging. Ultimately, DPSZ was successfully applied to living cell imaging, demonstrating the capability for long-term reversible imaging of HOCl and an endoplasmic reticulum (ER) targeting tendency. Furthermore, it successfully monitored the progression of acetaminophen (APAP)-induced liver injury and the therapeutic process mediated by resveratrol. This chemosensor provides an effective tool for the early diagnosis and treatment of DILI, further highlighting the critical role of HOCl in liver injury.

## Abstract 2

Iterative design of red-emitting fluorescent chemosensors for the reversible detection of HOCl.

## Graphical abstract

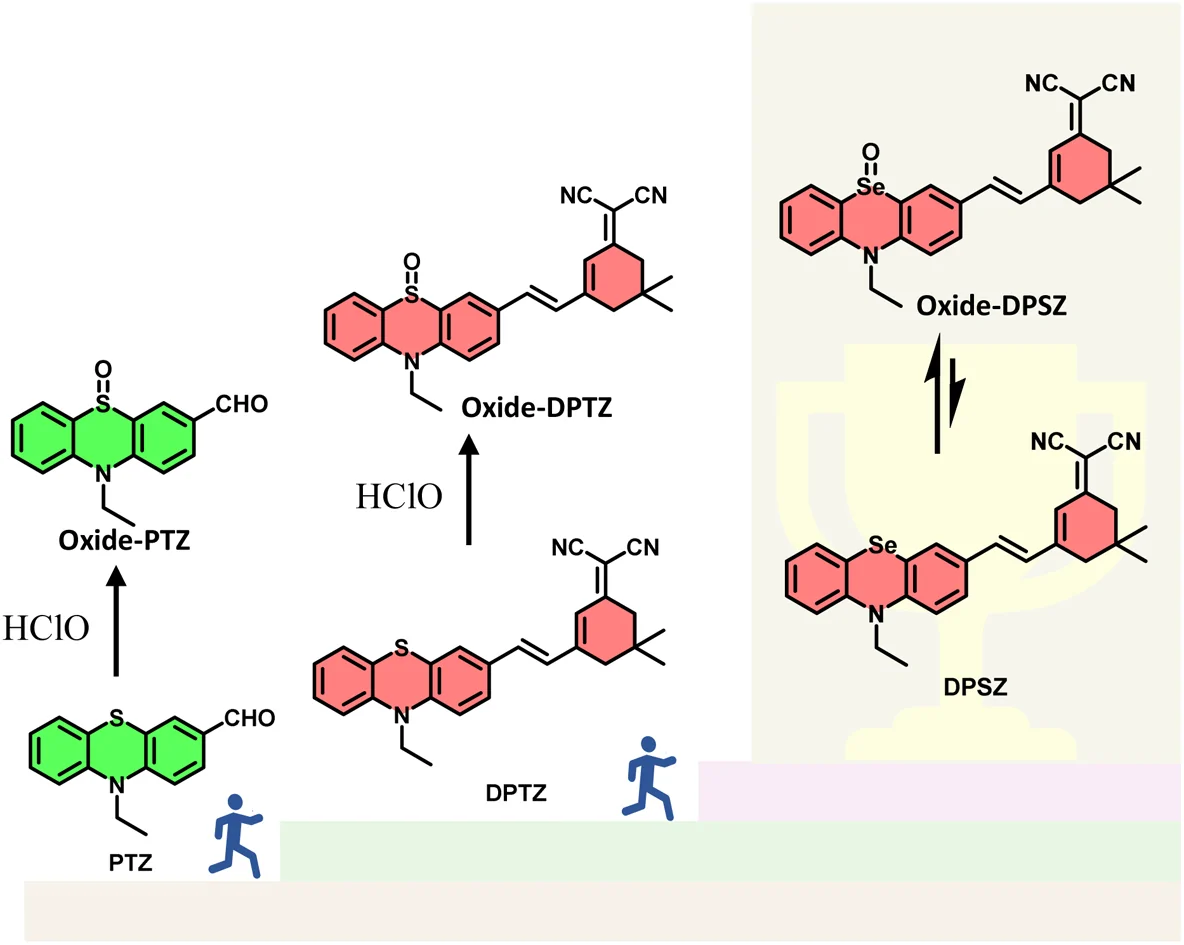

## Introduction

Hypochlorous acid/hypochlorite (HOCl/ClO^−^), as a core effector molecule of the human innate immune system, holds biological significance due to its dual role.^1^ Under physiological conditions, neutrophils directionally synthesize HOCl *via* the myeloperoxidase (MPO) system.^2,3^ As a potent oxidant, it efficiently eliminates pathogens. Its bactericidal mechanism is characterized by multiple targets: it penetrates microbial cell walls to oxidize protein sulfhydryl groups, thereby disrupting enzymatic active sites; it attacks nucleic acid bases, leading to genetic material fragmentation; and it alters cell membrane permeability, causing the leakage of intracellular contents. This broad-spectrum and efficient bactericidal capability establishes HOCl as the first chemical barrier of host defense.^4,5^

Under pathological conditions, the overproduction or impaired clearance of HOCl breaches the redox balance threshold, triggering oxidative stress injury. Its strong oxidizing property leads to host tissue damage, it modifies low-density lipoprotein to promote the formation of atherosclerotic plaques,^6^ and reacts with deoxyribonucleic acid (DNA) to generate chlorinated pyrimidines, inducing gene mutations.^7^ Recent studies further reveal that HOCl mediated protein chlorination plays a pivotal role in the progression of rheumatoid arthritis, neurodegenerative diseases, and cancer.^8^ Consequently, the detection of its specific biomarkers offers new avenues for early disease diagnosis.

Drug-induced liver injury (DILI) is a common adverse drug reaction in clinical practice and a critical cause of drug failure or adverse side effects.^9^ Its severity is manifested not only in the potential to induce acute liver failure, chronic liver disease, and even cirrhosis but also in its insidious clinical presentation and significant inter-individual variability, posing a serious threat to patient survival and medication safety.^10^ Currently, there is a lack of highly specific and sensitive diagnostic indicators.^11^ Conventional liver function tests often lag behind the onset of liver injury, making early warning difficult.^12^ Therefore, the discovery and validation of early biomarkers with tissue specificity and pathophysiological representativeness are of great significance. Such efforts are crucial for elucidating the mechanisms of DILI, achieving early recognition and risk stratification of injury, guiding rational clinical medication.

Within the complex pathological network of DILI, electrophilic groups or free radicals generated during drug metabolism can induce hepatocellular oxidative stress, recruit and activate neutrophils, and trigger the production of highly active HOCl.^13,14^ While HOCl participates in pathogen clearance, excessive amounts disrupt the redox balance, attacking hepatocyte membrane lipids, proteins, and mitochondrial DNA, thereby exacerbating hepatic inflammation and necrosis.^15^ Consequently, HOCl has emerged as a critical mediator linking immune activation to parenchymal cell injury.

Conventional liver function indicators, such as alanine aminotransferase (ALT) and aspartate aminotransferase (AST), lack sufficient sensitivity and specificity in the early stages of injury, making it difficult to accurately reflect the dynamics of immune-mediated oxidative damage.^16^ In contrast, HOCl generation is directly associated with MPO activity and the degree of inflammation. Given its specificity regarding pathophysiological mechanisms, HOCl possesses a strong theoretical basis as a novel biomarker.

To date, various analytical methods have indeed been established for the detection of HOCl, such as iodometry,^17^*N*,*N*-diethyl-*p*-phenylenediamine spectrophotometry,^18^ and electrochemical methods.^19^ However, these techniques are often invasive to biological samples, making accurate analysis in living systems challenging.^19^ In recent years, the development of highly selective and sensitive fluorescent chemosensor technologies has enabled real-time *in vivo* imaging and quantitative detection of HOCl.^20^ This provides technical support for elucidating its dynamic distribution and spatiotemporal evolution in DILI. Studies indicate that local hepatic HOCl levels rise significantly in DILI models, preceding changes in traditional biochemical markers, which highlights its potential value for early warning.^21^ Moreover, leveraging the chlorination and oxidation capabilities of HOCl, numerous strategies have been developed to respond to hypochlorous acid. These include the deprotection of *N*,*N*-dimethylthiocarbamates,^22^ the cleavage of imine bonds,^23^ the cleavage of the carbon–carbon double bond and,^25^ the hydrazone bonds,^24^ as well as the activation of methylene blue amides^25^ and the ring-opening of rhodamine lactams.^26^ These strategies achieve fluorescence variation by consuming HOCl, effectively converting chemical signals into optical signals for precise analysis, thereby significantly advancing the field of HOCl fluorescence sensing.

However, an undeniable fact is that the activation of these fluorescence signals inevitably entails the consumption of HOCl.^27,28^ Consequently, these chemosensors function dually as both sensors and scavengers. While this poses no significant issue in solvent testing or assays involving water and food samples, it presents a critical limitation for *in vivo* applications. Chemosensors based on this design strategy fail to meet the requirements for early diagnosis, as their capacity to scavenge HOCl may disrupt the homeostasis of the biological microenvironment and introduce potential toxicity. In fact, HOCl should not be detected as an isolated factor, as its concentration is governed by *in vivo* redox couples.^29,30^ Therefore, a rational HOCl chemosensor should be capable of responding simultaneously to both HOCl and reducing agents, thereby characterizing the fluctuations of endogenous HOCl with high fidelity.

**Scheme 1:**
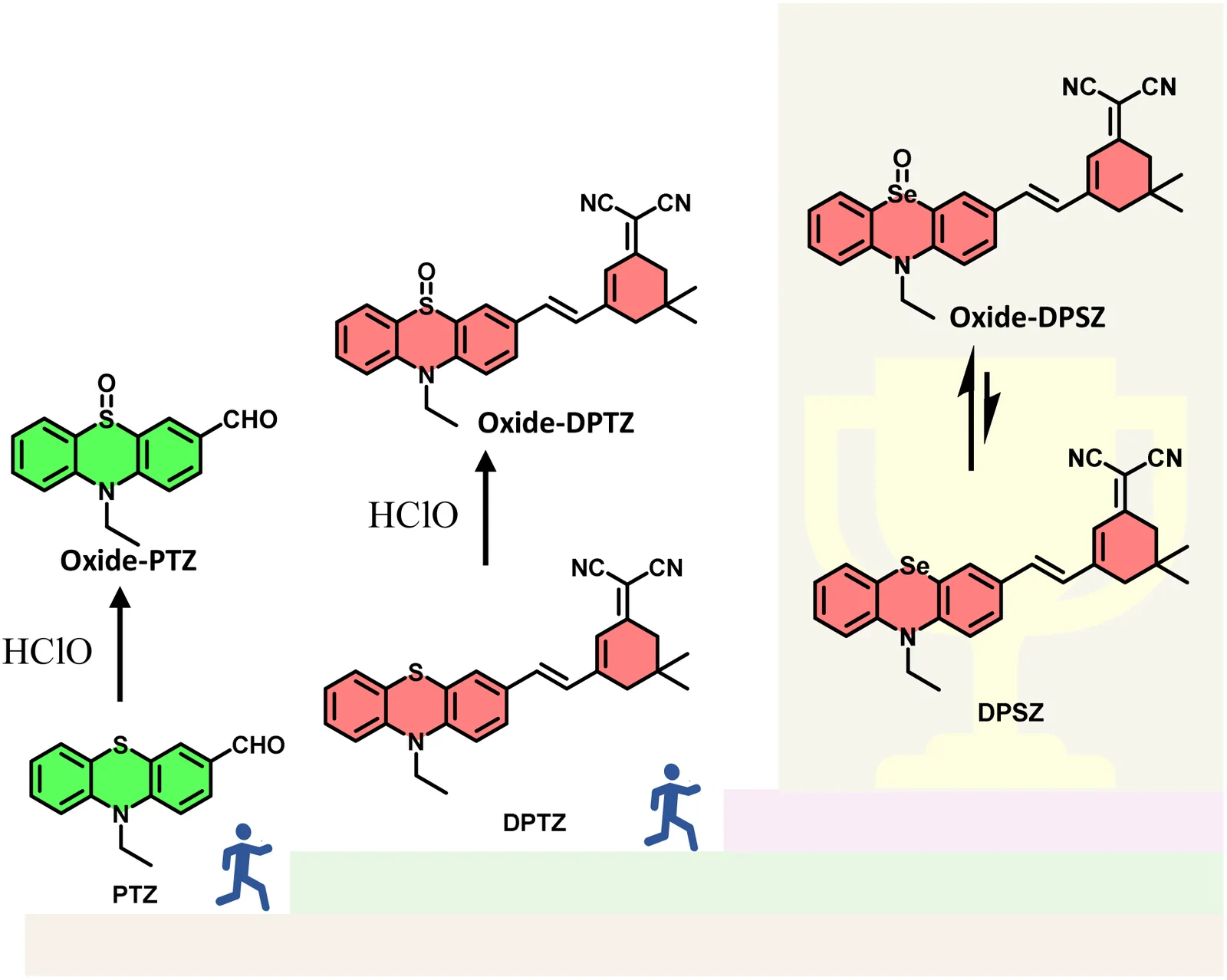
Iterative design of red-emitting fluorescent chemosensors for the reversible detection of HOCl.

In this work, we synthesized a fluorescent chemosensor PTZ utilizing phenothiazine as responsive moieties, which is capable of responding to the HOCl. In a test system containing only HOCl, PTZ was oxidized by HOCl to form S=O bonds, inducing a change in fluorescence. Furthermore, *via* a Knoevenagel condensation reaction, dicyanoisophorone (DCI) was reacted with phenoselenazine to enhance the intramolecular charge transfer (ICT) effect and extend the emission wavelength, yielding a red-emitting fluorescent chemosensor DPTZ. Finally, by replaced the S atom of DPTZ with a Se atom *via de novo* synthesis to yield DPSZ, the red emission and HOCl response were retained, while endowing the molecule with the capability for reversible response to the HOCl/GSH redox couple (Scheme 1). This chemosensor was successfully applied to the monitoring of treatment and progression in liver injury.

## Materials and methods

### General information and reagents

Unless otherwise indicated, all chemicals and organic solvents were obtained from commercial suppliers and used as received. The SI provide exhaustive descriptions of the instrumentation employed, test solution preparation, spectral analysis, nuclear Magnetic Resonance Spectroscopy (NMR) and high-resolution mass spectrometry (HRMS).

### Synthesis route

**Fig. 1:**
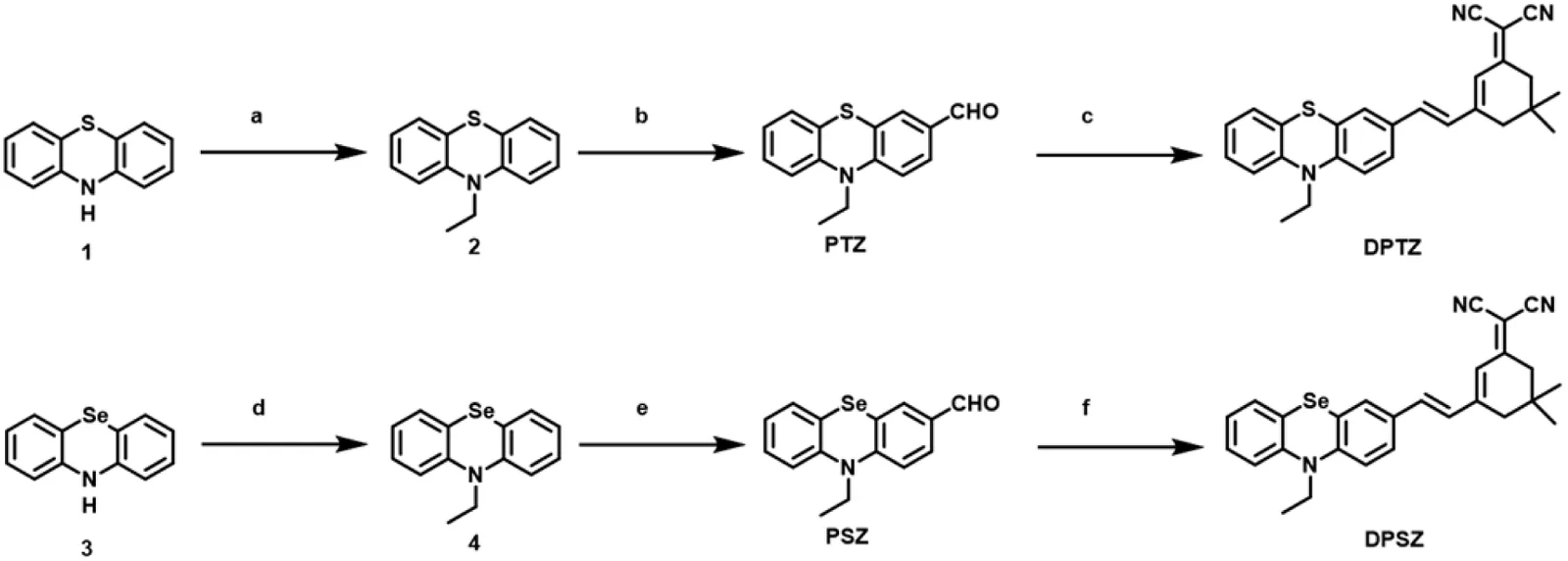
Synthetic route of PTZ, DPTZ, PSZ, DPSZ. (a) and (d) NaOH, bromoethane, DMSO, R.T., overnight. (b) and (e) *N*-methylformamide, POCl_3_, DCE, 85 °C, 6 h. (c) and (f) dicyanoisophorone, piperidine, acetonitrile, reflux, 8 h.

The overall synthetic route is shown in Fig. 1.

Synthesis of compound 2: phenothiazine (1 g, 5 mmol) and sodium hydroxide (NaOH, 1.6 g, 40 mmol) were added to dimethyl sulfoxide (DMSO, 13 mL), and the mixture was stirred in the dark for 1 hour. Bromoethane (570 mg, 5.2 mmol) was then added dropwise to the solution, and the reaction was stirred at room temperature overnight. Upon completion, the mixture was poured into water (400 mL) and extracted three times with dichloromethane (DCM). The combined organic layers were dried over anhydrous magnesium sulfate (MgSO_4_), filtered, and concentrated under reduced pressure. The crude product was purified by silica gel column chromatography (eluent: DCM/petroleum ether, 1 : 5 v/v) to afford compound 2 a white solid (913 mg, 81% yield). The characterization data matched well with the previous literature.^30,31^

Synthesis of compound PTZ: compound 2 (900 mg, 4 mmol) was dissolved in 1,2-dichloroethane (DCE, 10 mL). Under a nitrogen atmosphere at room temperature, a mixture of *N*-methylformamide (0.5 mL, 4.8 mmol) and phosphoryl chloride (POCl_3_, 0.54 mL, 6 mmol) was stirred for 30 min. The resulting mixture was then added dropwise slowly to the solution of compound 2, and the reaction was stirred at 85 °C for 6 h under a nitrogen atmosphere. After completion, the reaction mixture was cooled to room temperature, carefully poured into ice-water, and extracted three times with DCM. The combined organic layers were dried over anhydrous sodium sulfate (Na_2_SO_4_), filtered, and concentrated under reduced pressure. The crude product was purified by silica gel column chromatography (eluent: petroleum ether/ethyl acetate, 1 : 5 v/v) to afford PTZ as a yellow solid (600 mg, 59% yield). The characterization data matched well with the previous literature.^30,31^

Synthesis of compound DPTZ: PTZ (255 mg, 1 mmol) was dissolved in 10 mL of anhydrous acetonitrile, followed by the addition of piperidine (10 µL) and dicyanoisophorone (186 mg, 1 mmol). The mixture was refluxed for 8 h under a nitrogen atmosphere. After the solvent was removed, the crude product was purified by silica gel column chromatography (eluent: DCM/methanol, 50 : 1 v/v) to afford a reddish-brown solid, DPTZ (298 mg, 71% yield) ^1^H NMR (400 MHz,CDCl_3_) *δ* 7.23 (d, *J* = 2.0 Hz, 1H), 7.18–7.14 (m, 1H), 7.12 (dd, *J* = 7.4, 1.5 Hz, 1H), 6.93 (d, *J* = 6.7 Hz, 1H), 6.89 (d, *J* = 9.7 Hz, 2H), 6.87–6.83 (m, 2H), 6.82 (t, *J* = 3.4 Hz, 1H), 6.79 (s, 1H), 3.97–3.91 (m, 2H), 2.58 (s, 2H), 2.43 (s, 2H), 1.43 (t, *J* = 6.9 Hz, 4H), 1.07 (s, 6H). ^13^C NMR (100 MHz, CDCl_3_) *δ* 168.91, 153.88, 146.01, 143.52, 135.77, 129.75, 127.25, 127.18, 126.99, 126.93, 125.85, 124.46, 123.05, 122.70, 122.64, 115.00, 114.75, 113.53, 112.73, 42.77, 41.92, 39.00, 31.78, 27.81, 12.69. HRMS *m*/*z* calcd for C_27_H_26_N_3_S [M + H]^+^: 424.1769; found: 424.1849. Melt point: above 360 °C.

Synthesis of compound 4: compound 3 (1.23 g, 5 mmol) and NaOH (1.6 g, 40 mmol) were added to DMSO (18 mL), and the mixture was stirred in the dark for 1 h. Bromoethane (570 mg, 5.2 mmol) was then added dropwise to the solution, and the reaction was stirred at room temperature overnight. Upon completion, the mixture was poured into water (500 mL) and extracted three times with DCM. The combined organic layers were dried over anhydrous MgSO_4_, filtered, and concentrated under reduced pressure. The crude product was purified by silica gel column chromatography (eluent: DCM/petroleum ether, 1 : 3 v/v) to afford compound 4 a white solid (1.01 g, 74% yield).^32^

Synthesis of compound PSZ: compound 4 (825 mg, 3 mmol) was dissolved in DCE (10 mL). Under a nitrogen atmosphere at room temperature, a mixture of *N*-methylformamide (0.38 mL, 3.6 mmol) and phosphoryl chloride (POCl_3_, 0.4 mL, 4.5 mmol) was stirred for 30 min. The resulting mixture was then added dropwise slowly to the solution of compound 4, and the reaction was stirred at 85 °C for 6 h under a nitrogen atmosphere. After completion, the reaction mixture was cooled to room temperature, carefully poured into ice-water, and extracted three times with DCM. The combined organic layers were dried over anhydrous Na_2_SO_4_, filtered, and concentrated under reduced pressure. The crude product was purified by silica gel column chromatography (eluent: petroleum ether/ethyl acetate, 1 : 5 v/v) to afford PSZ as a yellow solid (666 mg, 73% yield). ^1^H NMR (500 MHz, CDCl_3_) *δ* 9.82 (s, 1H), 7.79 (d, *J* = 1.9 Hz, 1H), 7.70 (dd, *J* = 8.4, 1.9 Hz, 1H), 7.31 (dd, *J* = 7.8, 1.4 Hz, 1H), 7.27–7.22 (m, 1H), 7.03–6.97 (m, 3H), 4.02 (q, *J* = 7.0 Hz, 2H), 1.40 (t, *J* = 7.0 Hz, 3H). ^13^C NMR (125 MHz) *δ* 190.19, 151.05, 143.71, 131.54, 131.38, 130.17, 130.02, 127.85, 124.08, 122.01, 120.87, 117.40, 116.33, 43.53, 13.54. HRMS *m*/*z* calcd for C_15_H_14_NOSe [M + H]^+^: 304.0162; found: 304.0243. Melt point: 154.1–155.3 °C.

Synthesis of compound DPSZ: PSZ (303 mg, 1 mmol) was dissolved in 10 mL of anhydrous acetonitrile, followed by the addition of piperidine (10 µL) and dicyanoisophorone (186 mg, 1 mmol). The mixture was refluxed for 8 h under a nitrogen atmosphere. After the solvent was removed, the crude product was purified by silica gel column chromatography (eluent: DCM/methanol, 50 : 1 v/v) to afford a reddish-brown solid, DPSZ (296 mg, 63% yield). ^1^H NMR (400 MHz, CDCl_3_) *δ* 7.46 (d, *J* = 2.0 Hz, 1H), 7.34 (td, *J* = 8.7, 1.8 Hz, 2H), 7.25–7.19 (m, 1H), 7.00–6.95 (m, 3H), 6.94–6.92 (m, 1H), 6.85 (d, *J* = 16.1 Hz, 1H), 6.80 (s, 1H), 3.99 (q, *J* = 7.1 Hz, 2H), 2.58 (s, 2H), 2.43 (s, 2H), 1.39 (t, *J* = 6.9 Hz, 3H), 1.07 (s, 6H). ^13^C NMR (100 MHz, CDCl_3_) *δ* 167.20, 152.11, 144.95, 142.49, 134.06, 128.63, 128.15, 125.76, 125.49, 125.27, 121.54, 121.02, 120.42, 119.08, 115.10, 114.86, 111.76, 110.96, 41.24, 41.04, 37.26, 30.05, 26.08, 11.62. HRMS *m*/*z* calcd for C_27_H_26_N_3_Se [M + H]^+^:472.1214; found: 472.1294. Melt point: above 360 °C.

### *In vitro* assay of the fluorescent chemosensor

To evaluate the response, 10 µM of PTZ, DPTZ, or DPSZ was incubated with HOCl at concentrations ranging from 0 to 100 µM in phosphate buffered saline (PBS) buffer (10 mM, pH 7.4; 3 mL total volume). After a reaction time of 10 minutes, the samples were analyzed. The assay solution was prepared with a final *N*,*N*-dimethylformamide (DMF) content of 10%.

### Cell viability and fluorescence imaging experiments with cells

HepG2 cells were purchased from Wuhan Procell Life Science & Technology Co., Ltd (Procell, Wuhan, China), cultured in Dulbecco's modified Eagle's medium (DMEM) high glucose medium supplemented with 10% fetal bovine serum and 1% penicillin/streptomycin at 37 °C under 5% CO_2_ atmosphere.

The 3-(4,5-dimethyl-2-thiazolyl)-2,5-diphenyl-2*H*-tetrazolium bromide (MTT) assay was applied to measure the cell viability. After the different concentration of DPSZ (0, 1.25, 2.5, 5, 10, 20 µM) treatment, 50 µL of MTT solution at 1 : 5 dilution was added to each well and cells were incubated for additional 4 h at 37 °C. The culture media were then removed and the formazan dye was dissolved in DMSO. Absorbance was measured at 490 nm with a microplate reader. The cell viability was measured and normalized to the vehicle group. The mean optical density (OD) of 6 wells in each group was used to calculate cell viability as follows: cell viability = OD (group to be tested)/OD (control group) × 100%.

Prior to confocal imaging, living HepG2 cells were collected and reseeded onto 15 mm glass-bottom dishes, allowing 24 h for adherence. DPSZ (10 µM) was treated the cell for 30 min at 37 °C. Zeiss 880 NLO microscope was utilized for image acquisition. The fluorescent chemosensor was imaged *via* the red channel with an excitation wavelength of 488 nm and an emission range of 600–700 nm. Quantitative analysis involved calculating the average fluorescence intensity within defined regions of interest. Results shown are representative of five independent experiments and are expressed as mean ± S.D.

### Animal experiments

Six-week-old male C57BL/6 mice (20 ±2 g) were purchased from Hunan Slack Jingda Laboratory Animal Co., Ltd (Changsha, China), with the experimental animal production license No. SCXK (Xiang) 2024-0009. Mice were fasted overnight prior to administration. The study was divided into five groups. Group I (control) received an intraperitoneal (i.p.) injection of 100 µL saline (15 mg mL^−1^). Group II and Group III were administered APAP (300 mg kg^−1^ and 600 mg kg^−1^, respectively) *via* i.p. injection. Group IV and Group V were pretreated with Res (50 mg kg^−1^) 12 hours prior to the administration of APAP (300 mg kg^−1^ and 600 mg kg^−1^, respectively). One hour after the final treatment, all five groups of mice were intravenously (i.v.) injected with DPSZ (100 µM) for *in vivo* imaging. The excitation wavelength was set at 480 nm, and the emission was collected in the range of 600–700 nm. The animal experiment was approved by the Ethics Committee of Hengyang Medical College at the University of South China.

## Results and discussion

### Stepwise design of HOCl fluorescent chemosensor

**Fig. 2:**
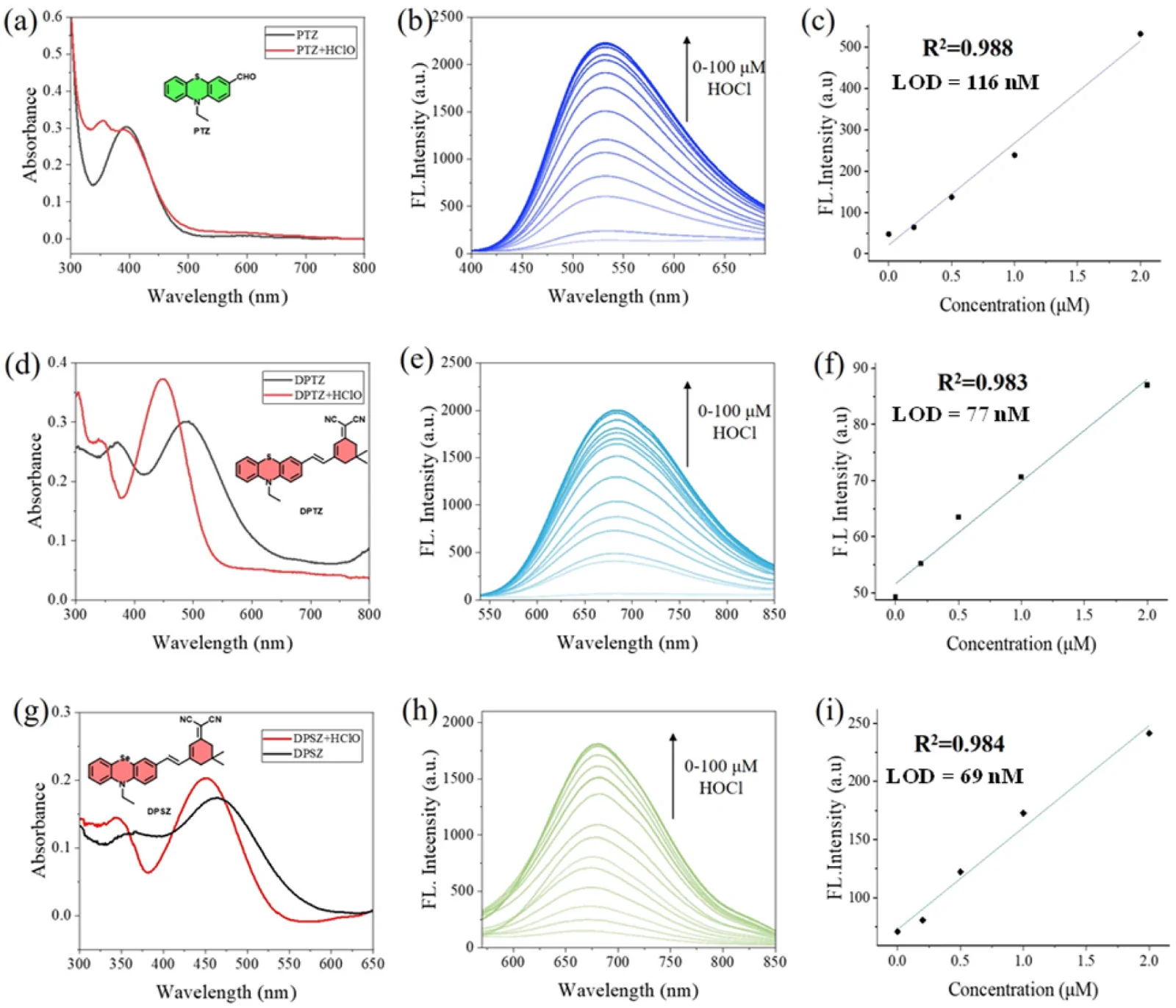
Changes of absorption of 10 µM of PTZ (a), DPTZ (d), DPSZ (g) after addition of HOCl (100 µM). Concentration dependent fluorescence changes of 10 µM of PTZ (b), DPTZ (e), DPSZ (h) after addition of HOCl (0–100 µM). Linear correlation between 10 µM of PTZ (c), DPTZ (f), DPSZ (i) and the HOCl concentration (0–2 µM). *λ*_ex_ = 356 nm for test of PTZ, *λ*_ex_ = 443 nm for test of DPTZ, *λ*_ex_ = 440 nm for test of DPSZ.

Initially, to achieve optical detection of HOCl, we synthesized a phenothiazine-based dye, PTZ, which relies on the responsiveness of a thioether moiety toward HOCl. Upon the addition of HOCl, the absorption spectrum of PTZ exhibited a significant change, the maximum absorption peak from 401 nm changed to 356 nm (Fig. 2a). The fluorescence intensity gradually increased with the addition of HOCl with a 16-fold enhancement (Fig. 2b). Meanwhile, the response of the fluorescent chemosensor PTZ to HOCl (0–2 µM) showed a good linear relationship (Fig. 2c), the limit of detection (LOD) was determined to be 116 nM demonstrating that PTZ possessed potential capability for sensing HOCl.

However, the short-wavelength emission of PTZ is susceptible to interference from biological components such as hemoglobin and does not fall within the optimal window for imaging. Therefore, we extended the conjugated system of PTZ by reacting the exposed aldehyde group with a strong electron acceptor, dicyanoisophorone (DCI), to obtain the red-emitting dye DPTZ. DPTZ exhibited an absorption wavelength of 496 nm. After reacted with HOCl, the absorption wavelength underwent a blue shift, and the maximum absorption reached 443 nm (Fig. 2d). The solution color changed significantly from reddish-brown to yellow with the naked eye (Fig. S1). The fluorescent intensity gradually enhanced through the addition of HOCl, the maximum peak emerged in 682 nm, placed the emission window within the favorable region for biological imaging (Fig. 2e). Moreover, the response between DPTZ and HOCl with a good linear relationship in the range of 0–2 µM (*R*^2^ = 0.983) (Fig. 2f). The LOD was determined to be 77 nM. Importantly, the Stokes shift reached 239 nm after DPSZ the responded to HOCl. There is almost no overlap between the absorption and emission spectra, which minimizes fluorescence self-absorption and background interference.

Finally, we noted that the electronegativity of Se is lower than that of S, we synthesized PSZ*de novo* by replacing S with Se, and subsequently reacted it with the DCI acceptor to synthesize DPSZ. The absorption peak of DPSZ was 470 nm, showing little difference from that of DPTZ. Upon exposure to HOCl, the absorption of DPSZ underwent a blue shift to 440 nm (Fig. 2g). The solution color changed significantly from pink to yellow with the naked eye (Fig. S2). The fluorescence intensity increased sharply at 688 nm, resulted in a final enhancement of 18-fold (Fig. 2h). Meanwhile, the response of DPSZ to HOCl (0–2 µM) showed a favorable linear relationship (Fig. 2i). The LOD was determined to be 69 nM. The above results indicate that all three chemosensors are capable of responding to HOCl with fluorescence change.

To further elucidate the underlying sensing mechanism, HRMS analysis was conducted. Upon treatment of DPSZ with HOCl, a prominent signal at *m*/*z* 510.1406 was detected (Fig. S3), which matches well with the calculated molecular weight of the oxidized product DPSZ ([M + Na]^+^ = 510.1163). As expected, the corresponding oxidation products of PTZ (Fig. S4) and DPTZ (Fig. S5) upon reaction with HOCl were also detected by HRMS. These results provide strong evidence for the oxidation process induced by HOCl.

### Optical response of DPSZ toward HOCl

**Fig. 3:**
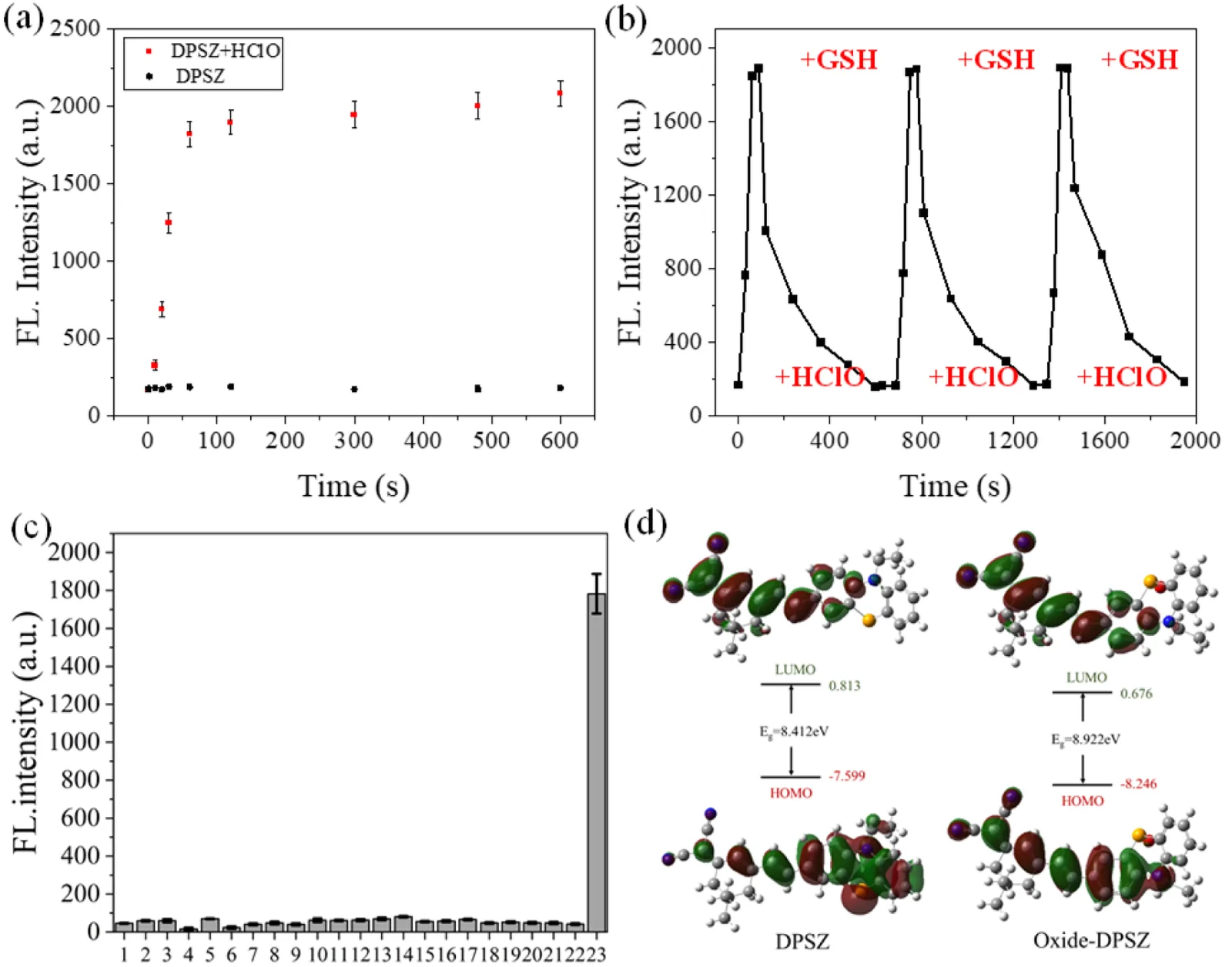
(a) Fluorescence intensity of DPSZ (10 µM) in the presence of HOCl (100 µM) concentrations as a function of reaction time. (b) Fluorescence intensity of DPSZ (10 µM) incubated with HOCl (100 µM) followed by GSH (120 µM). (c) The fluorescence intensity DPSZ (10 µM) in the presence of HOCl and other relevant species. 1, blank; 2, ONOO^−^; 3, H_2_O_2_; 4, O_2_^−^; 5, HO^.^; 6, Cys; 7, Hcy; 8, GSH; 9, H_2_S; 10, K^+^; 11, Na^+^; 12, Mg^2+^; 13, MPO; 14, NQO1; 15, NaClO_2_; 16, NaClO_4_; 17, DTT; 18, DTE; 19, DHLA; 20, NAC; 21, β-ME; 22, CoA; 23, HOCl. The concentrations of GSH, CoA, MPO, and NQO1 were set at 1 mM, 1 µM, 1 µM, and 1 µM, respectively, while all other analytes were at 100 µM. *λ*_ex_ = 440 nm, *λ*_ex_ = 680 nm, *n* = 3. (d) The HOMO/LUMO frontier molecular orbital calculated showing the S1 → S0 transition of DPSZ and the oxide product oxide-DPSZ.

Subsequently, we thoroughly evaluated the spectral properties of DPSZ. Upon exposure to 100 µM HOCl, the chemosensor reached response saturation within 1 min, with the fluorescence intensity remaining stable for 10 minutes. In contrast, negligible fluorescence variation was observed over 10 minutes in the absence of HOCl, demonstrating the extremely rapid response speed of DPSZ (Fig. 3a). More importantly, when 120 µM GSH was added 0.5 minutes after the reaction with HOCl, the fluorescence intensity gradually decreased over 10 minutes, returning to the level of the free chemosensor. This HOCl/GSH redox cycle was repeated three times, validating our hypothesis regarding the reversible detection of HOCl (Fig. 3b). Furthermore, the response of the oxidized DPSZ to GSH exhibited a benign linear relationship (*R*^2^ = 0.997) (Fig. S6).

To investigate whether DPSZ functions under complex physiological or pathological conditions, we examined its fluorescence response in the PBS buffer with varying pH values. As shown in Fig. S7, in the presence of HOCl, DPSZ exhibited high fluorescence intensity at 688 nm across the pH range of 3.0 to 10.0, with deviations from the intensity at pH 7.4 not exceeding 10%. Upon exposure to GSH, the fluorescence intensity reverted to that of the free chemosensor and remained largely unaffected by pH, indicating that DPSZ can monitor the HOCl/GSH redox pair in physiological environments.

To evaluate selectivity, the responses between DPSZ with other relevant species were tested, including reactive chlorine species (NaClO_2_, NaClO_4_), oxidants (ONOO^−^, H_2_O_2_, KO_2_, HO˙), reductants (Cys, Hcy, GSH, H_2_S, DL-dithiothreitol (DTT), Dithioerythritol (DTE), dihydrolipoic acid (DHLA), *N*-acetylcysteine (NAC), β-mercaptoethanol (β-ME)), ions (K^+^, Na^+^, Mg^2+^), and enzymes (MPO, NQO1). As illustrated in Fig. 2c, only HOCl induced a significant fluorescence increase, while all other species caused minimal changes. Meanwhile, during evaluation of potential interference in the reductive process, strong reductants such as GSH, DTT, DTE, DHLA, NAC and β-ME restored the original fluorescence signal (Fig. S8), considering that GSH is the most abundant non-protein thiol in biological systems and occupies the central position in the cellular redox network, we maintain that GSH plays the dominant role in the reduction process within living systems. These results demonstrate that DPSZ selectively monitors the HOCl/GSH redox pair with high specificity.

Ultimately, the energy changes associated with the HOCl response of DPSZ were uncovered by density functional theory (DFT) calculations. Oxide-DPSZ and DPSZ occurred at the LOMO → HOMO energy levels were calculated to 8.922 eV and 8.412 eV, respectively (Fig. 3d). Compared to DPSZ, Oxide-DPSZ had a slightly stronger push–pull electronic effect, thus triggered an off-on fluorescent change.

In conclusion, the above results demonstrate the potential of DPSZ respond to HOCl in complex biological environments.

### Cytotoxicity and fluorescence imaging

Given that the unique reversible detection capability of DPSZ for HOCl has been established *in vitro*, the next step is to apply it at the cellular level to validate its potential for biological applications. The MTT assay was initially employed to evaluate the biocompatibility of DPSZ. Even after 24 hours incubation of HepG2 cells with 20 µM DPSZ, cell death remained below 15%, indicating minimal cytotoxicity of the chemosensor (Fig. S9).

To assess the ability of DPSZ to detect intracellular HOCl fluctuations in living cells, we conducted confocal microscopy experiments using four distinct cellular groups. Group i (control) was incubated with DPSZ alone for 30 minutes. Group ii was pretreated with lipopolysaccharide (LPS) for 12 hours to stimulate endogenous HOCl production *via* immune activation, followed by DPSZ incubation.^33^ Group iii received LPS pretreatment for 12 hours, then exposure to the HOCl scavenger Tiron for 30 minutes prior to DPSZ addition. Group iv was incubated with Tiron alone without LPS stimulation. Confocal imaging revealed markedly enhanced fluorescence intensity in the red channel for Group ii compared to Group i, indicating HOCl detection. In Group iii, fluorescence intensity decreased significantly relative to Group II due to Tiron-mediated HOCl clearance. Group iv exhibited fluorescence levels similar to Group i.^34^ These results demonstrate that DPSZ reliably monitors dynamic changes in intracellular HOCl concentration through its fluorescence response, supporting its utility for biological applications.

**Fig. 4:**
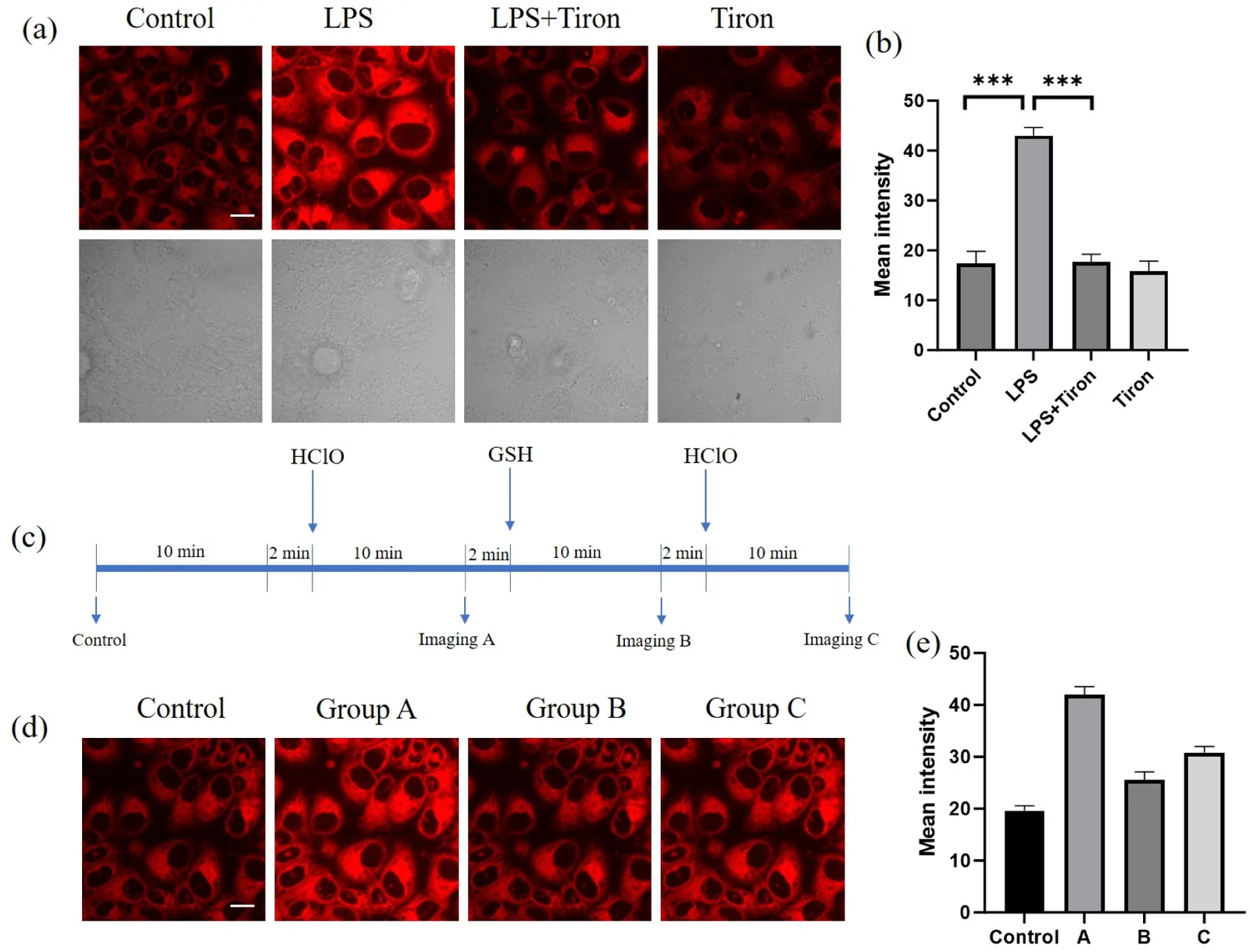
(a) Living cell imaging of the chemosensor DPSZ (10 µM) responding to endogenous HOCl in HepG2 cells. From top to bottom: red channel, bright field. From left to right: control, LPS (1 µg mL^−1^) treated for 12 h, LPS (1 µg mL^−1^) pretreated for 12 h and then Tiron (10 µM) for 1 h, Tiron (10 µM) for 1 h (b) relative fluorescence intensity of living cell imaging in (a). (c) Schematic illustration of the reversible cycle detection of the HOCl/GSH redox couple in living cells. (d) Living cell imaging of the chemosensor DPSZ (10 µM) responding to HOCl (10 µM) and GSH (20 µM) in HepG2 cells. (e) Relative fluorescence intensity of living cell imaging in (d). Scale bar = 10 µm. *λ*_em_ = 600–750 nm, *λ*_ex_ = 488 nm, *n* = 3.

Next, we further validated the unique reversible response of DPSZ to HOCl. After adding DPSZ to HepG2 cells, 10 µM HOCl was subsequently introduced, and time-lapse imaging was performed every 2 minutes under a confocal microscope. The red fluorescence intensity progressively increased due to continuous oxidation of DPSZ by HOCl. At the 10-minute timepoint, GSH (20 µM) was added to the cells, and imaging was continued. Notably, the cellular fluorescence intensity gradually decreased thereafter. These results demonstrate that DPSZ dynamically reports intracellular HOCl levels in response to redox equilibrium fluctuations (Fig. 4).

### Cellular colocalization of DPSZ

**Fig. 5:**
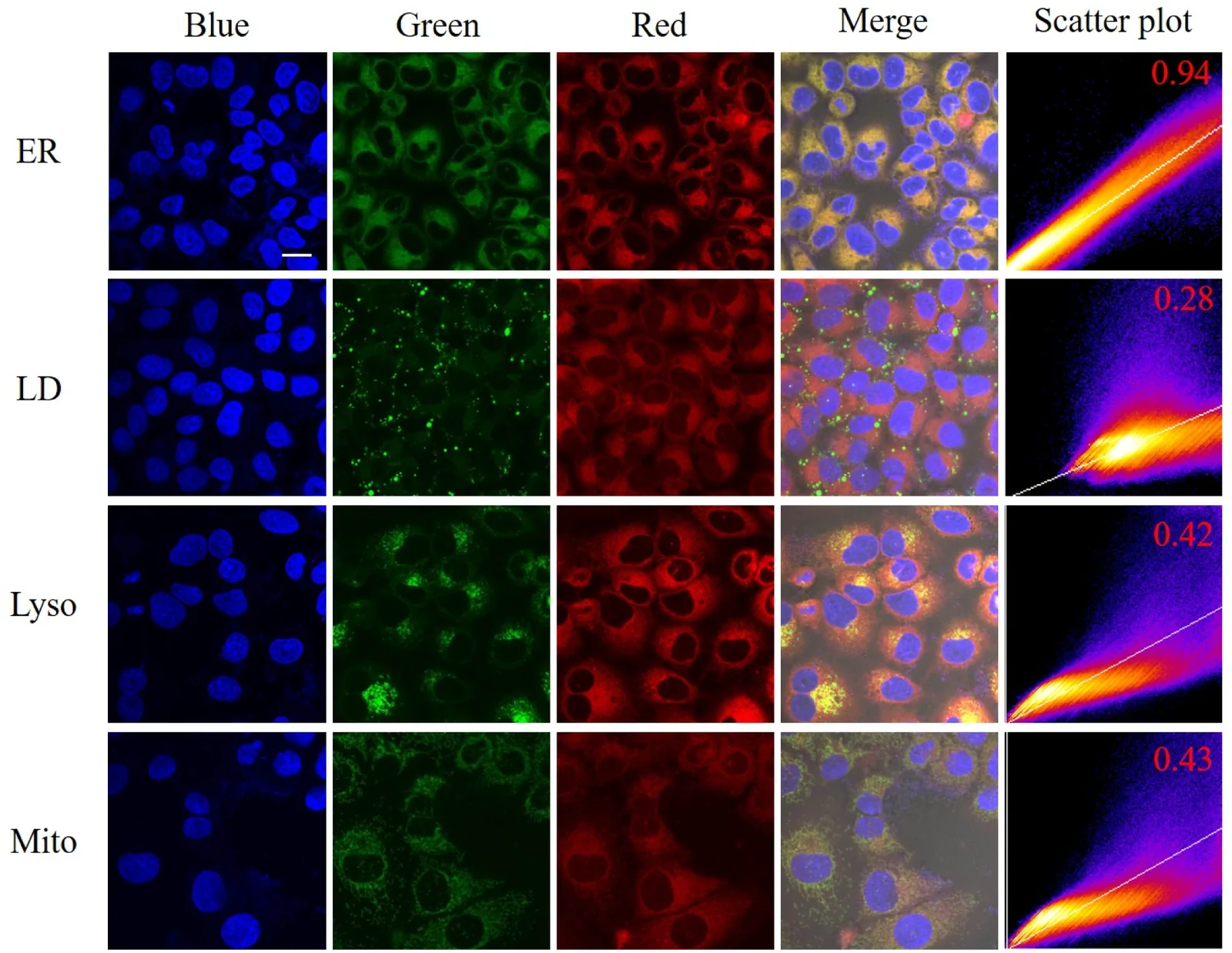
Co-localization imaging of DPSZ, from left to right: blue channel, green channel, red channel, merge of three fluorescent channel and bright field, scatter plot, from top to bottom:, endoplasmic reticulum, lipid droplet, lysosome, mitochondria. Red channel: *λ*_em_ = 600–700 nm, *λ*_ex_ = 488 nm. Green channel: *λ*_em_ = 500–550 nm, *λ*_ex_ = 488 nm. Especially, nucleus tracker: *λ*_em_ = 420–460 nm, *λ*_ex_ = 405 nm. Scale bar = 10 µm. Chemosensor DPSZ (10 µM) pretreated with LPS (2 µg mL^−1^) for 12 h, concentration of each tracker was 0.1 µM.

In fluorescence imaging experiments, we observed that the fluorescence signal exhibited a reticular pattern filling the cytoplasm. Therefore, we further investigated the subcellular targeting preference of DPSZ by performing co-staining with commercial green organelle-specific fluorescent dyes as positive controls. The results showed that the co-localization coefficient of DPSZ with the endoplasmic reticulum (ER) was 0.94, while those with other organelles were mitochondria (Mito, 0.43), lysosomes (Lyso, 0.42) and lipid droplets (LD, 0.28). Previous literature has indeed indicated that fluorescent dyes with a DCI structure tend to target the endoplasmic reticulum due to appropriate lipophilicity, which may also explain the high co-localization coefficient of DPSZ with the ER.^35,36^ Thus, DPSZ has the potential to monitor redox changes on the endoplasmic reticulum (Fig. 5).

Based on DPSZ's endoplasmic reticulum-targeting property in hepatocytes and its reversible detection capability for HOCl, we further validated its potential as a novel diagnostic tool for liver injury. Acetaminophen (APAP), a classic anti-inflammatory drug, is metabolized by cytochrome P450 enzymes to produce the highly reactive toxic intermediate *N*-acetyl-*p*-benzoquinone imine (NAPQI). This depletes GSH, induces endoplasmic reticulum stress, and ultimately causes irreversible liver damage.^37^

**Fig. 6:**
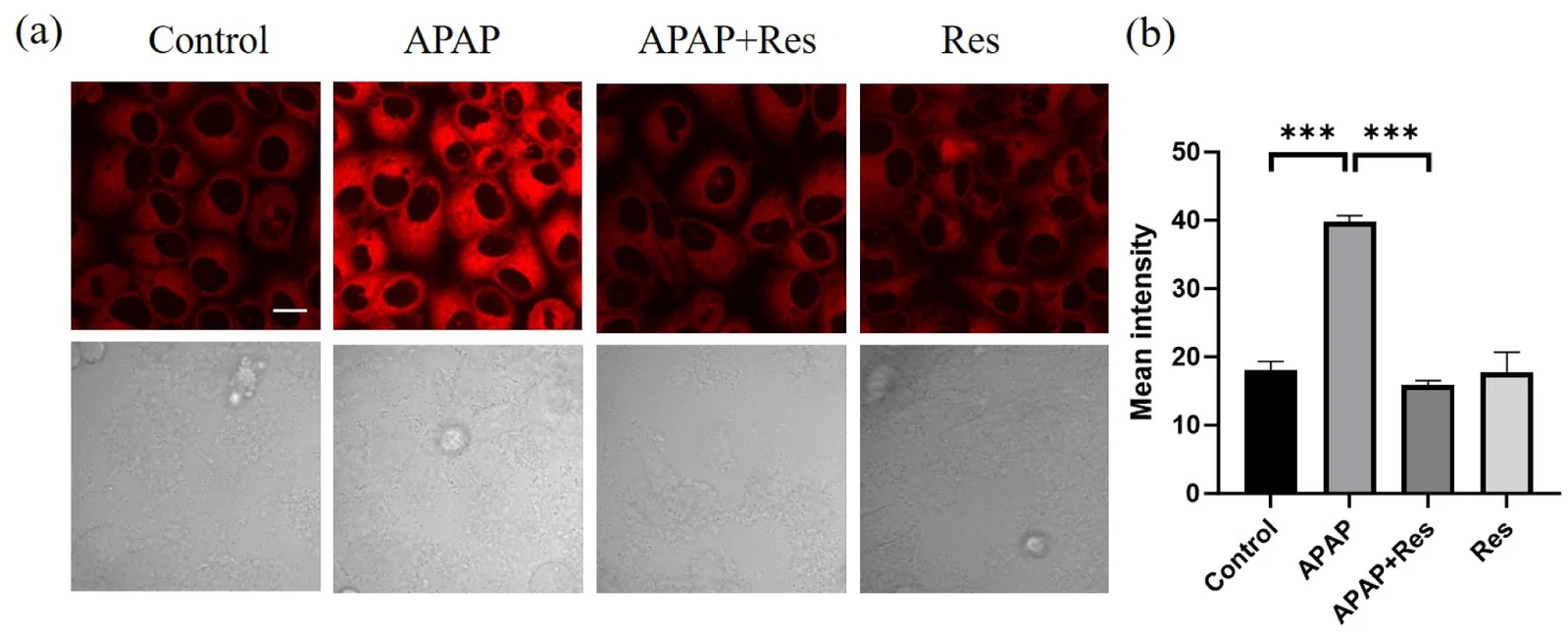
(a) Living cell imaging of the chemosensor DPSZ (10 µM) responding to endogenous HOCl in HepG2 cells. From top to bottom: red channel, bright field. From left to right: control, APAP (10 µM) treated for 12 h, APAP (10 µM) pretreated for 12 h and then Res (10 µM) for 1 h, Res (10 µM) for 1 h (b) relative fluorescence intensity of living cell imaging in (a).

We incubated HepG2 cells with DPSZ and observed the effects of different APAP doses, finding that red fluorescence intensity progressively increased with higher doses (Fig. 6). Upon adding the natural compound resveratrol, fluorescence intensity showed a decreasing trend. Therefore, HOCl as an early indicator of oxidative stress-induced injury, holds promise as a biomarker for early diagnosis of liver damage progression. Meanwhile, resveratrol, functioning as an antioxidant molecule with additional therapeutic effects, demonstrates potential for development as a treatment for liver injury.^38^

### *In vivo* imaging for APAP-induced liver injury

**Fig. 7:**
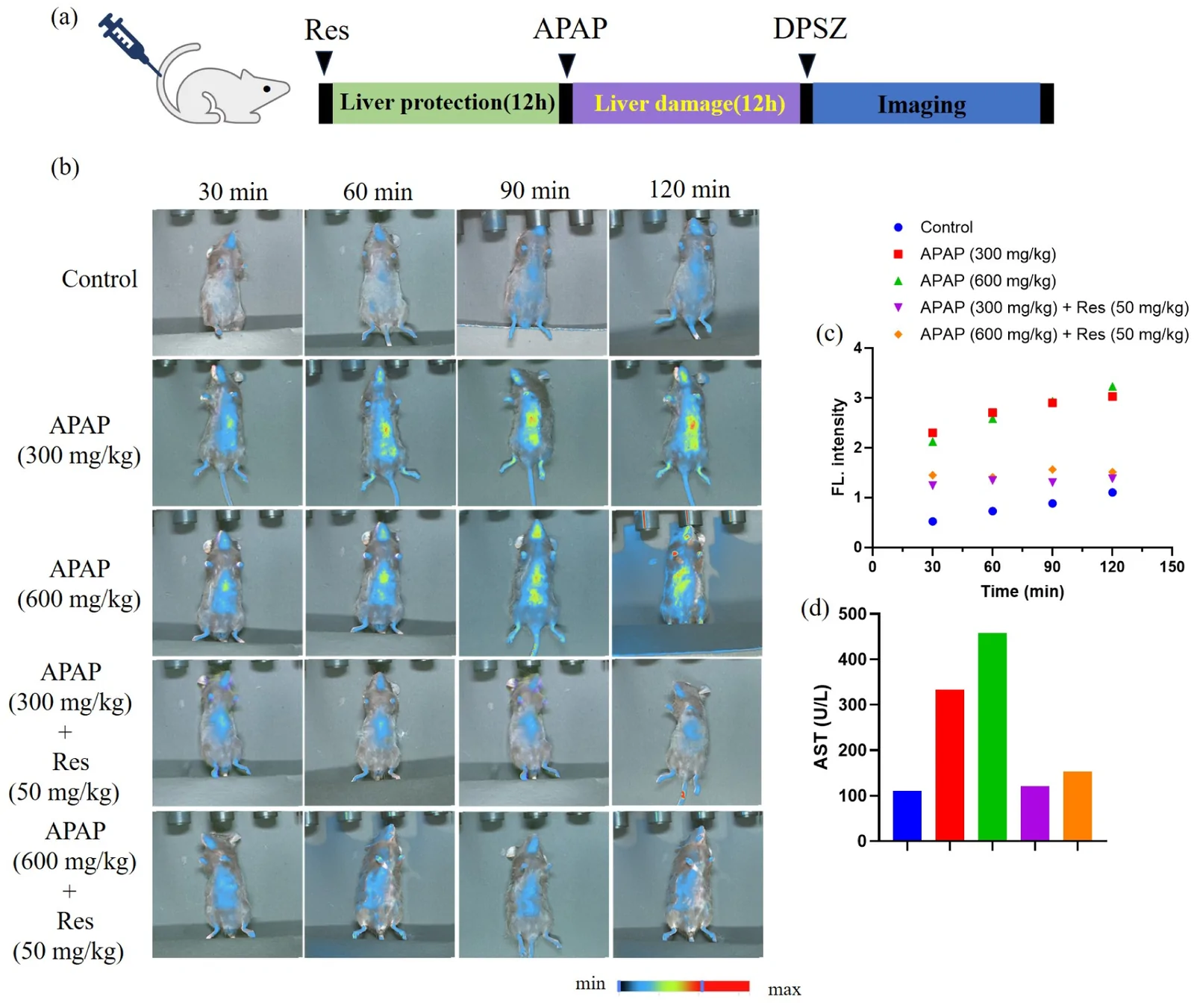
(a) Diagrammatic illustration of the therapeutic scheme for APAP and Res treatment. (b) Fluorescence imaging of the DPSZ responding to HOCl in the APAP-induced liver injury. *λ*_ex_ = 488 nm. *λ*_em_ = 610–650 nm. (c) Relative fluorescence intensity of (a). (d) Biochemical markers (AST) of liver injury across different treatment groups.

Finally, we established an APAP-induced model of drug-induced liver injury to evaluate the diagnostic capability of DPSZ for liver damage in live mice. 12 Hours after intraperitoneal injection of APAP, mice were intravenously injected with DPSZ*via* the tail vein, followed by time-dependent imaging every 30 minutes. Res has emerged as a promising candidate drug against liver injury due to its potent abilities to scavenge free radicals, activate nuclear factor erythroid 2-related factor 2 (Nrf2), and suppress inflammatory responses. Res was administered *via* intraperitoneal injection 12 hours prior to APAP treatment. *In vivo* imaging results demonstrated that hepatic fluorescence intensity progressively increased over time. This elevation was significantly more pronounced in mice treated with 600 mg kg^−1^ APAP. In contrast, both Res-treated groups exhibited a notable reduction in fluorescence intensity. Serum AST analysis across the five groups revealed that while normal mice had an AST level of 110 U L^−1^, APAP-treated mice reached 334 U L^−1^ and 458 U L^−1^, respectively. In the Res-protected groups, serum AST levels recovered to 121 U L^−1^ and 154 U L^−1^, indicating a certain degree of alleviation of liver injury. Meanwhile, as shown in Fig. S10, treatment with different concentrations of APAP significantly increased MPO levels in the liver, while administration of Res inhibited this elevation, partially indicating that the observed increase in HOCl concentration in mouse liver was attributable to heightened MPO activity (Fig. 7). These findings suggest that DPSZ provides non-invasive, real-time diagnosis and holds potential as a diagnostic tool for drug-induced liver injury.

## Conclusions

In this work, we sequentially synthesized three fluorescent molecules to achieve high-fidelity visualization of HOCl centric redox fluctuations during liver injury. Initially, PTZ successfully detected hypochlorite in solution, exhibiting a 16-fold increase in fluorescence intensity. Subsequently, DPTZ was synthesized by conjugating PTZ with the strong electron-withdrawing DCI group. DPTZ inherited the responsiveness to hypochlorite while offering the advantages of near-infrared emission and a large Stokes shift. Furthermore, DPSZ was synthesized *de novo* by replacing the S atom in the DPTZ skeleton with Se, endowing it with the capability for long-term and reversible HOCl imaging. Ultimately, DPSZ was applied to cellular and murine liver injury models, demonstrating excellent ER targeting and real-time HOCl monitoring capabilities. Leveraging DPSZ, we successfully visualized the HOCl burst associated with APAP-induced hepatocellular injury and drug-induced liver injury in mice, while also validating the antioxidant and hepatoprotective effects of Res. In summary, DPSZ holds great promise as a novel and suitable diagnostic tool for liver injury.

## Author contributions

C. L. designed the study and the experiments, collected data and were responsible for the interpretation. C. L., C. Z., F. H. and W. K. drafted the manuscript. All authors have read and agreed to the published version of the manuscript.

## Conflicts of interest

There are no conflicts to declare.

## Supplementary Material

RA-016-D6RA04366F-s001

## Data Availability

The original contributions presented in this study are included in the article/supplementary information (SI). Further inquiries can be directed to the corresponding authors. Supplementary information: general methods, NMR and MS spectra, the absorption and emission spectra, cell cytotoxicity experiments. See DOI: https://doi.org/10.1039/d6ra04366f.
