# Supplementary material for: Stepwise design and synthesis of red-emitting, reversible, HOCl chemosensors for monitoring the progression of drug-induced liver injury
Source: RSC Adv. 2026 Jul 28;16(39):43552–9. doi: 10.1039/d6ra04366f (PMC13411160; doi:10.1039/d6ra04366f)
Supplement: Supplementary file 1 [file RA-016-D6RA04366F-s001.pdf]

Supplementary Information (SI) for RSC Advances.
This journal is © The Royal Society of Chemistry 2026

Stepwise Design and Synthesis of Red-Emitting, Reversible, HOCl Chemosensors for Monitoring the Progression of Drug-Induced Liver Injury

Experimental section

Instruments and materials.

All chemicals and solvents are commercially available and used without further purification. TLC plate was used to monitor the reaction and detected by an ultraviolet lamp. Compounds were purified by a silica chromatography column using the indicated solvent mixture. ^{1}H and ^{13}C NMR spectra were recorded on AVANCE NEO 400 or AVANCE NEO 500. Fluorescence spectra were collected with a Hitachi F-7100 fluorescence spectrophotometer. Absorption spectra were determined on Tech comp UV2600-vis spectrometer. Cells were cultured in a CO_2 incubator (FORMA STERI-CYCLE i160, Thermo Fisher Scienfitic). High-resolution mass spectra (HRMS) were obtained from DIONEX UltiMate 3000 & Bruker Compact TOF mass spectrometer. Fluorescence imaging was conducted with confocal laser scanning microscopy (CLSM, Zeiss LMS880). All aqueous solutions were prepared by using ultrapure water with a resistivity of 18.25 MΩ (purified by Milli-Q system supplied by Millipore). The pH of the buffer solution was controlled by a digital pH meter (FE20, MettlerToledo).

Structure	Max (ex/em)	Stokes shift	Organelle target	Animal model	Ref.
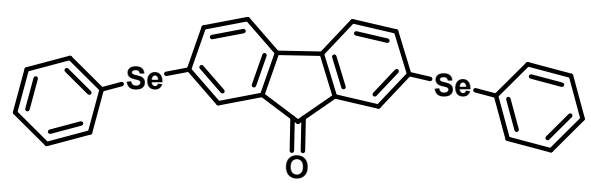	415/520	105	-	-	*Chem Commun* 2015; 51; 50:10150-10153.
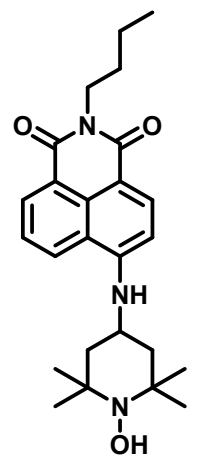	445/530	85	-	-	*Spectrochim Acta A Mol Biomol Spectrosc.* 2021;247:119138.
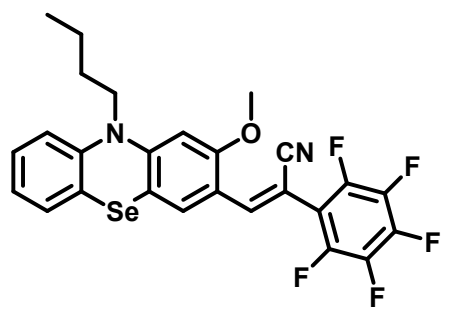	350/480	130	Lipid droplets	PD mouse	*Anal. Chem.* 2026; 98; 1: 809-817
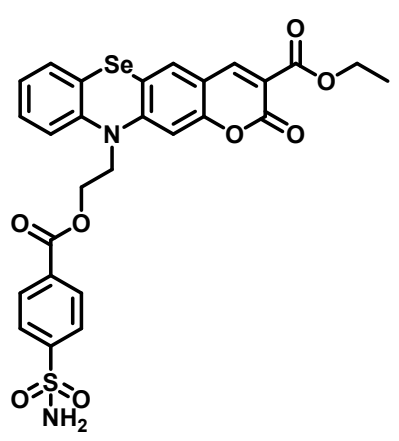	390/514	124	Golgi body	Acute lung lnjury mouse	*Anal. Chem.* 2023; 95; 20: 8002-8010
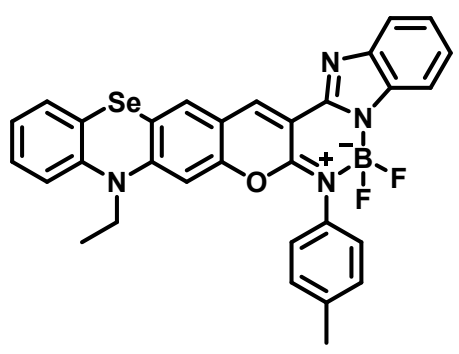	450/555	105	-	Wilson's disease mouse	*Chem Commun* 2025;61;29:5507-5510.
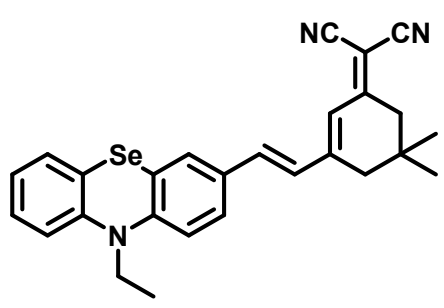	440/688	248	ER	APAP-induced Liver injury mouse	This work

Table S1 Overview and comparison of selected spectral properties and biological applications of reversible HOCl-responsive fluorescent probes.

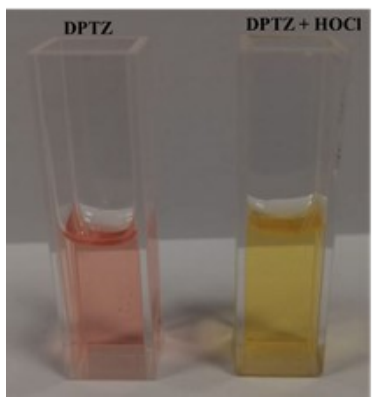

Fig. S1 Macroscopic changes of **DPTZ** solution before and after response to HOCl.

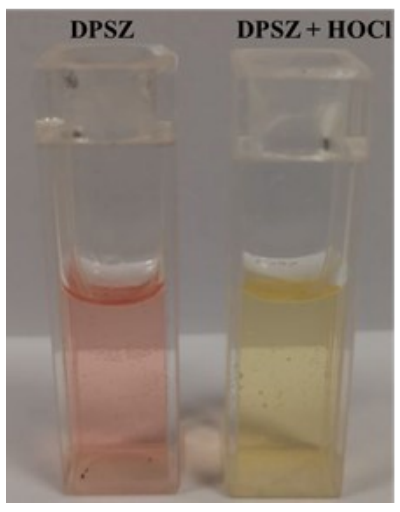

Fig. S2 Macroscopic changes of **DPSZ** solution before and after response to HOCl.

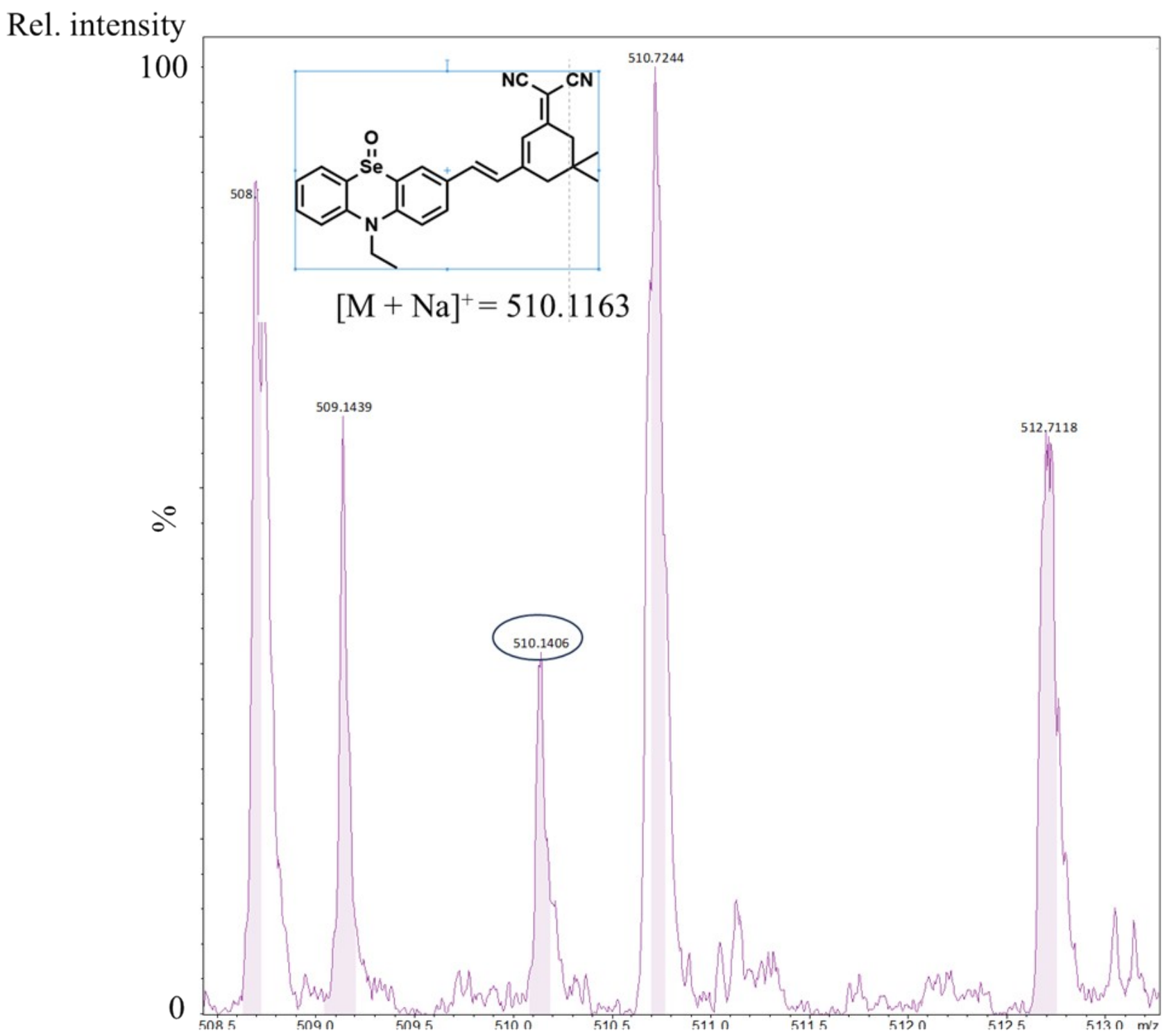

Figure S3. Mass spectra of **DPSZ** with HOCl.

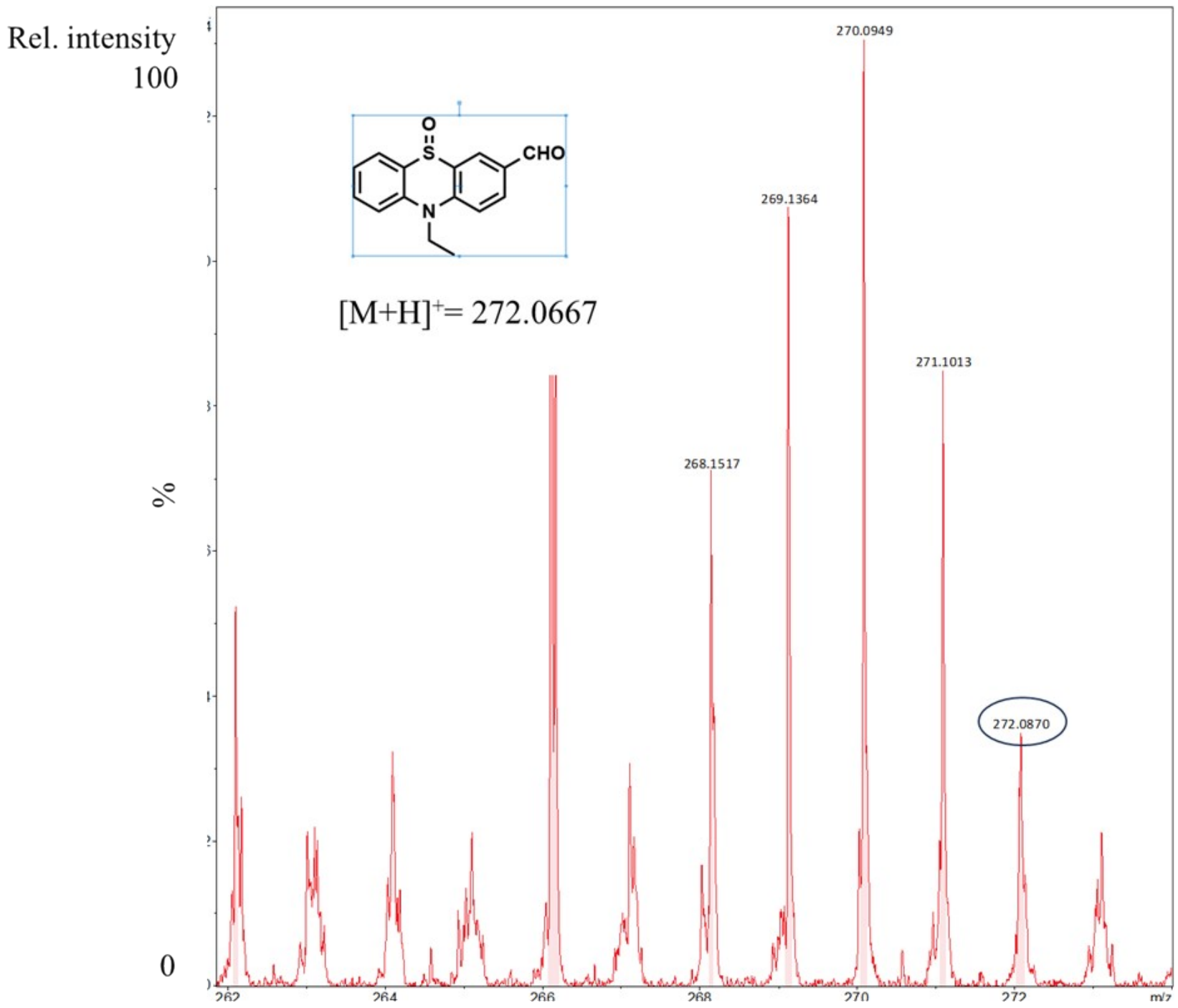

Figure S4. Mass spectra of **PTZ** with HOCl.

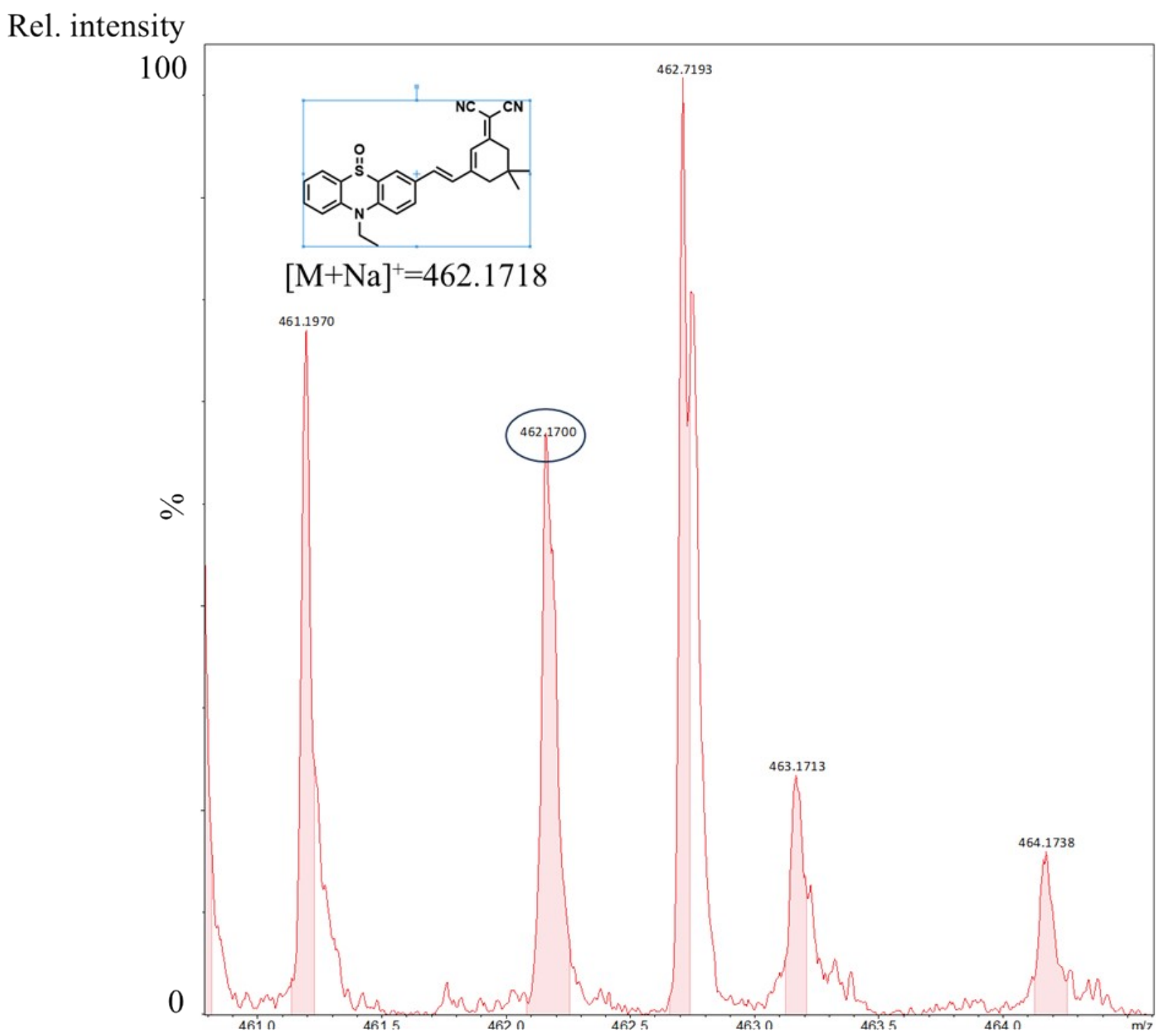

Figure S5. Mass spectra of **DPTZ** with HOCl.

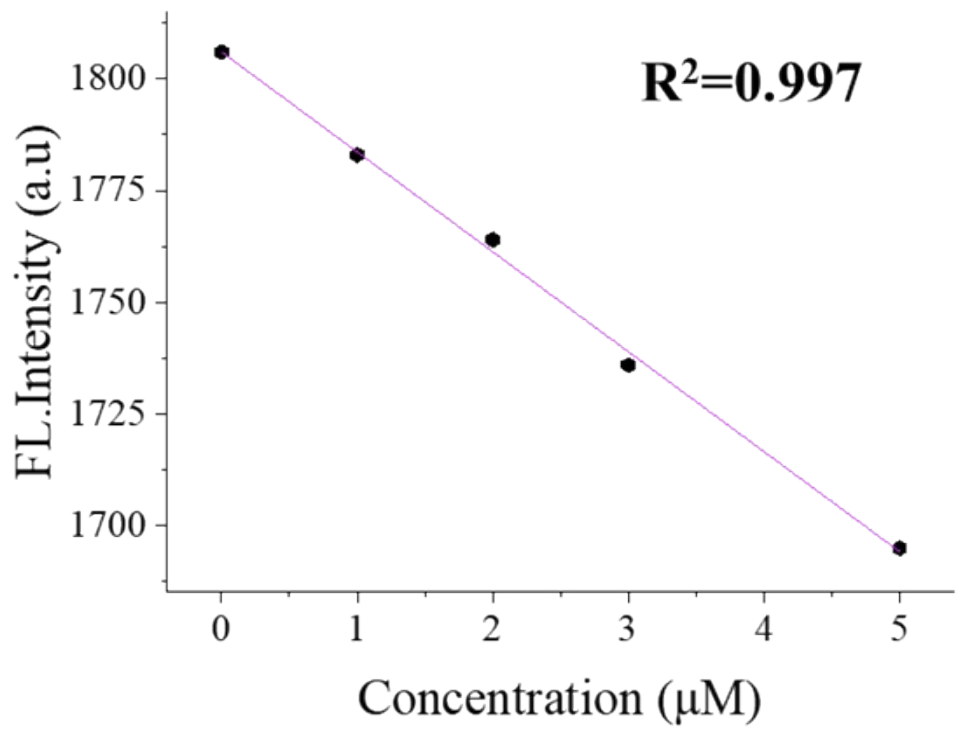

Figure S6. Linear correlation between 10 μM of **DPSZ** reacted with HOCl (100 μM) and then reacted with GSH (0-5 μM). λex = 440 nm.

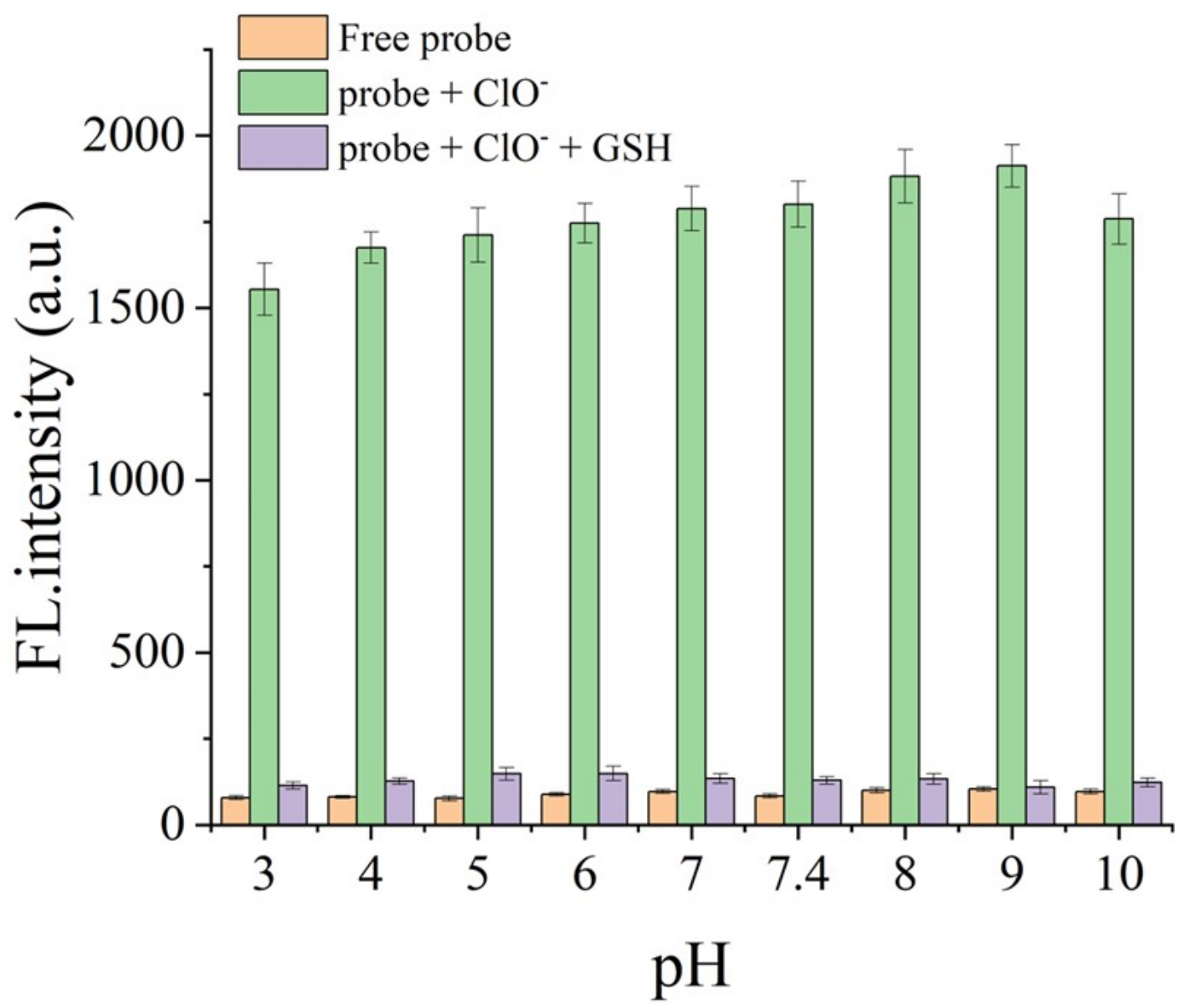

Figure S7. Effect of pH on the fluorescence (at 663 nm) of **DPSZ** (10 μM) in the absence and presence of HOCl (100 μM) and presence of HOCl and GSH (120 μM). λex = 440 nm. n = 3.

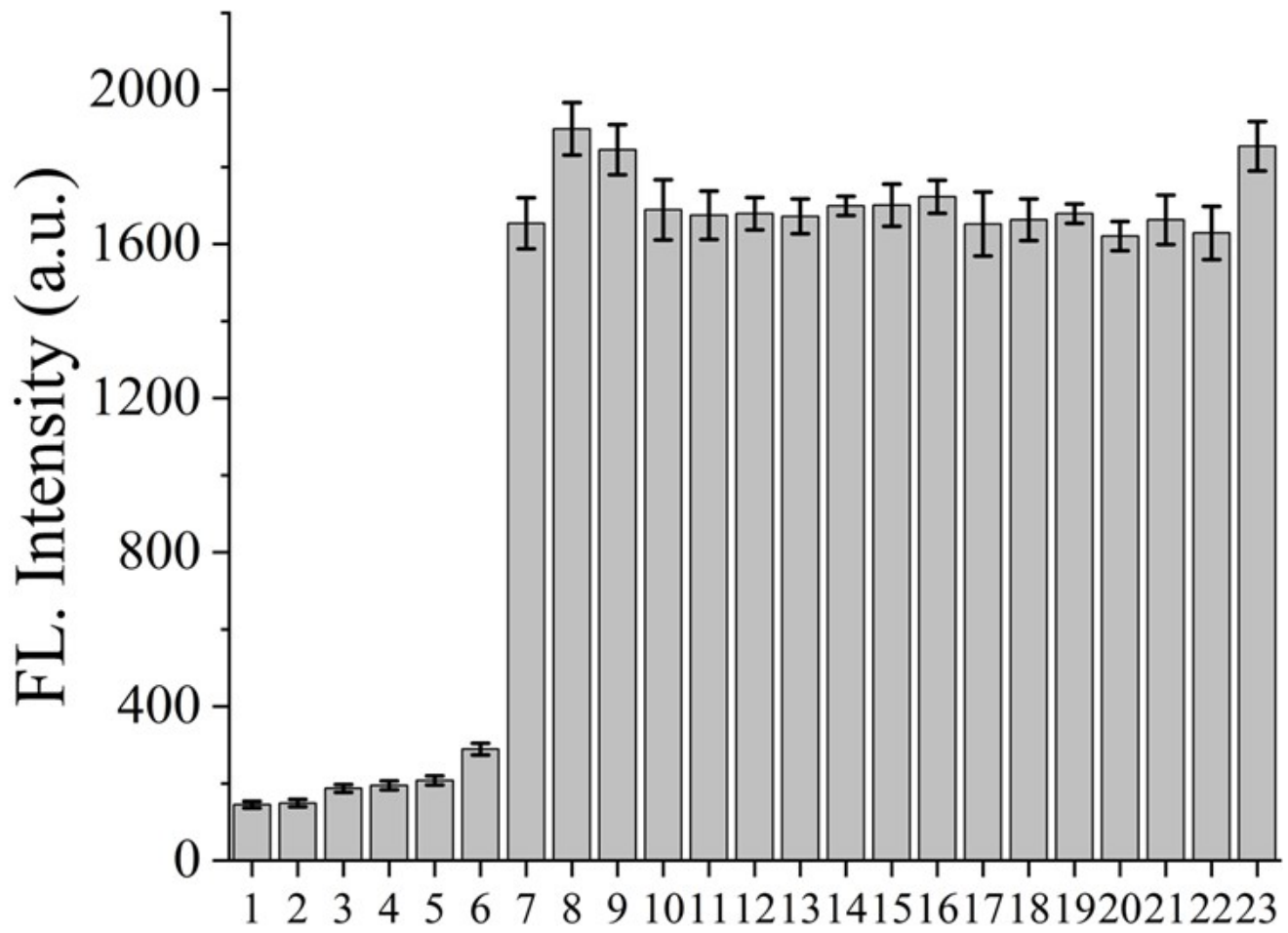

Fig. S8 The fluorescence intensity **DPSZ** (10 μM) in the presence of HOCl (100 μM) and then reacted with and other relevant species. 1, GSH; 2, DTT; 3, DTE; 4, DHLA; 5, NAC; 6, β-ME; 7, CoA; 8, $NaClO_2$; 9, $NaClO_4$; 10, $ONOO^-$; 11, H_2O_2; 12, $O_2^{\cdot-}$; 13, HO·; 14, Cys; 15, Hcy; 16, GSH; 17, H_2S; 18, K^+; 19, Na^+; 20, Mg^{2+}; 21, MPO; 22, NQO1; 23, HOCl. The concentrations of GSH, MPO, CoA, and NQO1 were set at 1 mM, 1 μM, 1 μM and 1 μM, respectively, while all other analytes were at 100 μM. λex = 440 nm, λex = 680 nm, n =3.

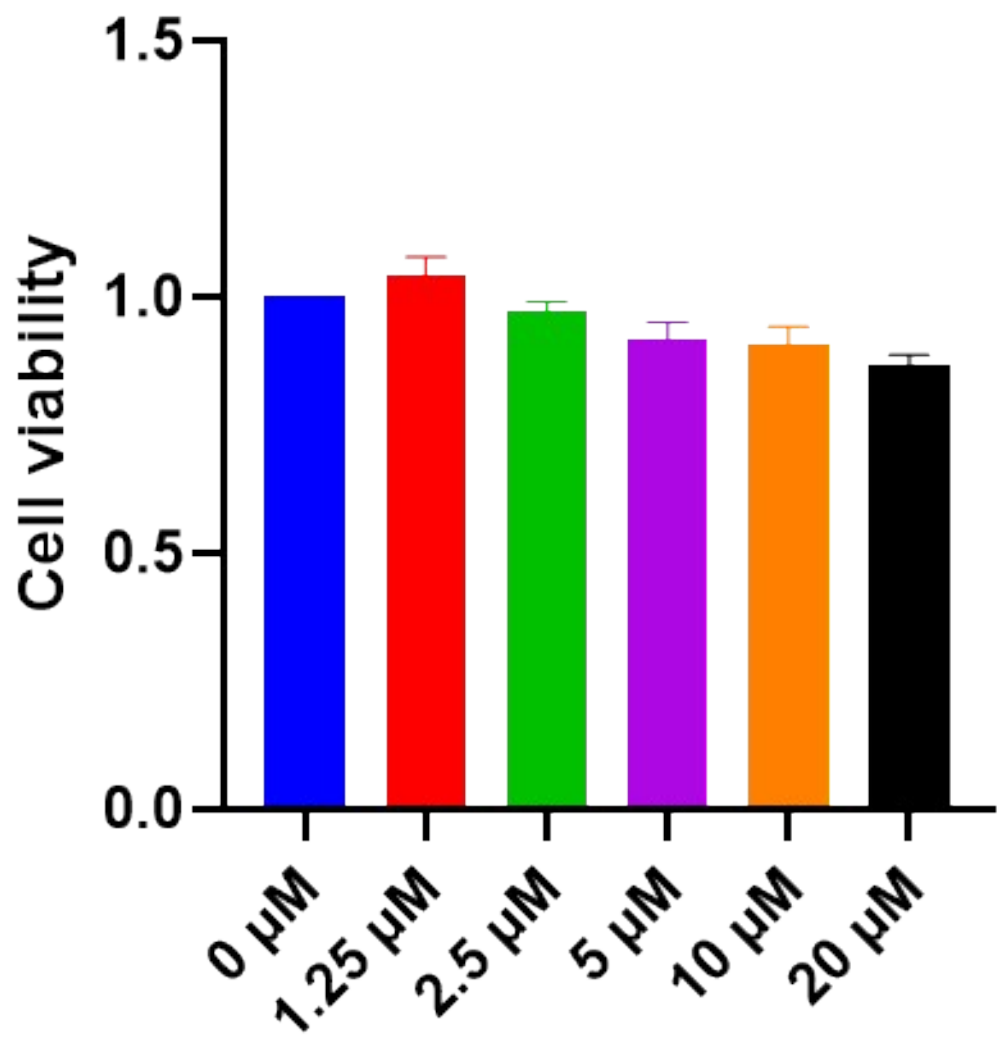

Fig. S9 Cytotoxicity of the **DPSZ** by MTT assay for 24 h treatment at different concentration (0, 1.25, 2.5, 5, 10, 20 μM). Cell viability (%) was calculated using following equation: Viability = (mean Abs. of treated wells/mean Abs. of control wells) ×100%

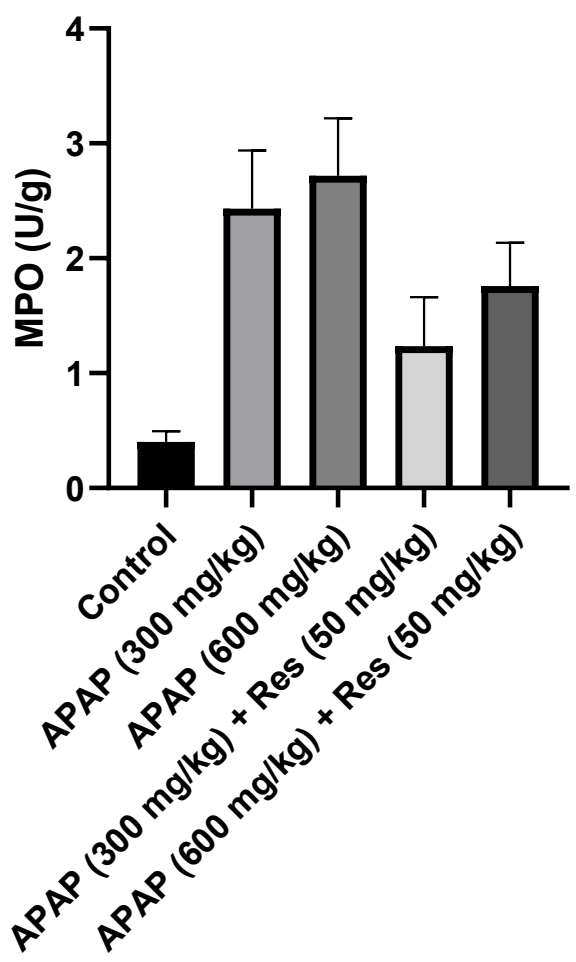

Fig. S10 The levels of MPO in livers of mice from all groups (n = 3). This value was measured using an MPO assay kit (Wuhan Elabscience Biotechnology Co., Ltd.). Mice were sacrificed 24 hours after drug administration ended. 0.1 g fresh liver tissue sample was excised, rinsed with ice-cold PBS (0.01 M, pH 7.4) at 4°C, blotted dry with filter paper, weighed, and placed into a homogenization tube. The tissue was homogenized with ice-cold assay buffer from the kit at a weight (g): volume (mL) ratio of 1:4 (equivalent to 2:8), followed by incubation at 37°C for 15 minutes. The optical density (OD) value was then measured at 460 nm using a microplate reader. MPO activity (U/g) = $\Delta A \times 0.175 \times 1000 / V_{sample} \times f$. ΔA: sample absorbance value minus control absorbance value, V_{sample}: volume of serum or plasma sample, f: dilution factor for samples that underwent no pretreatment.

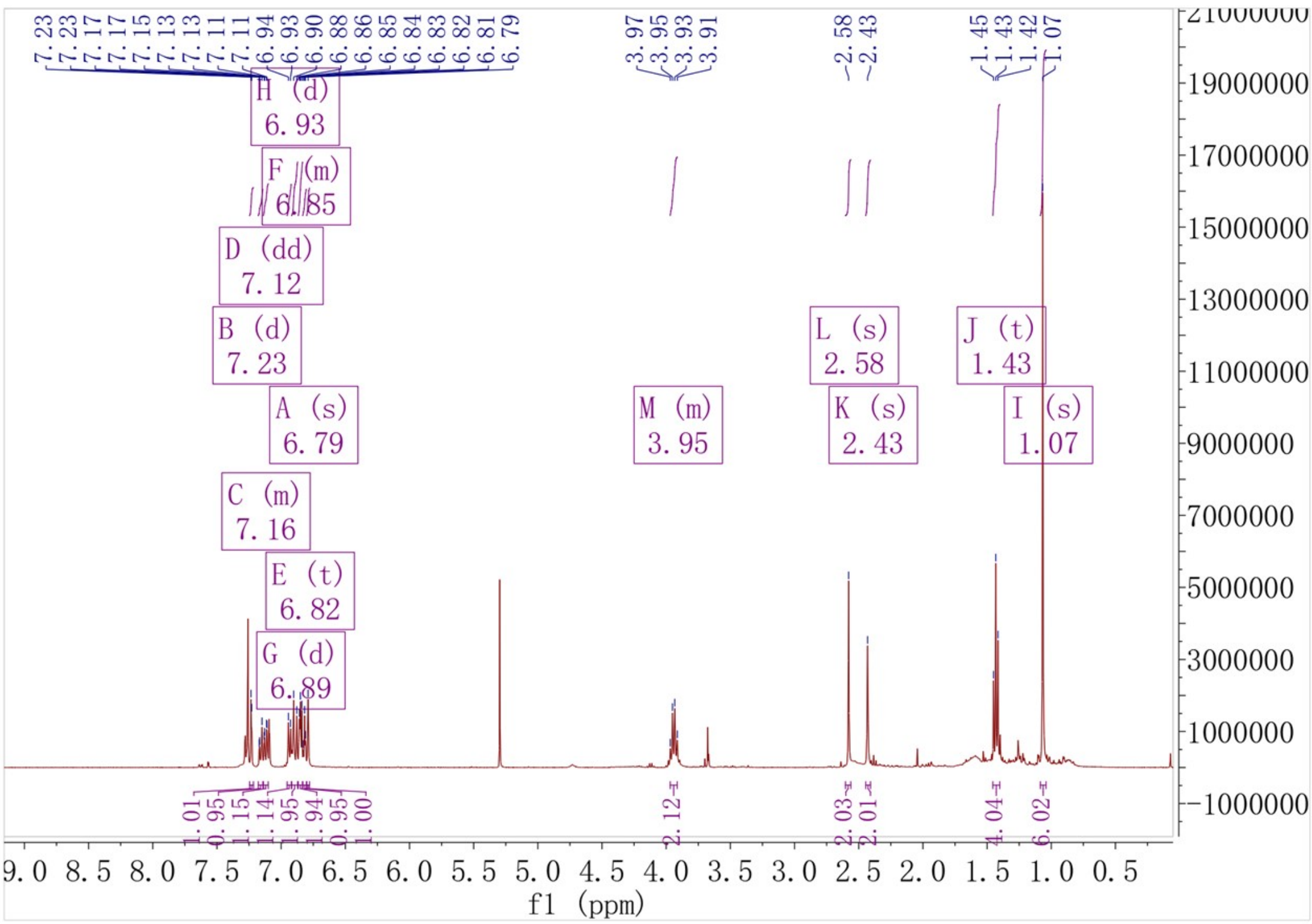

Fig. S11 ^{1}H NMR of **DPTZ** in $CDCl_3$

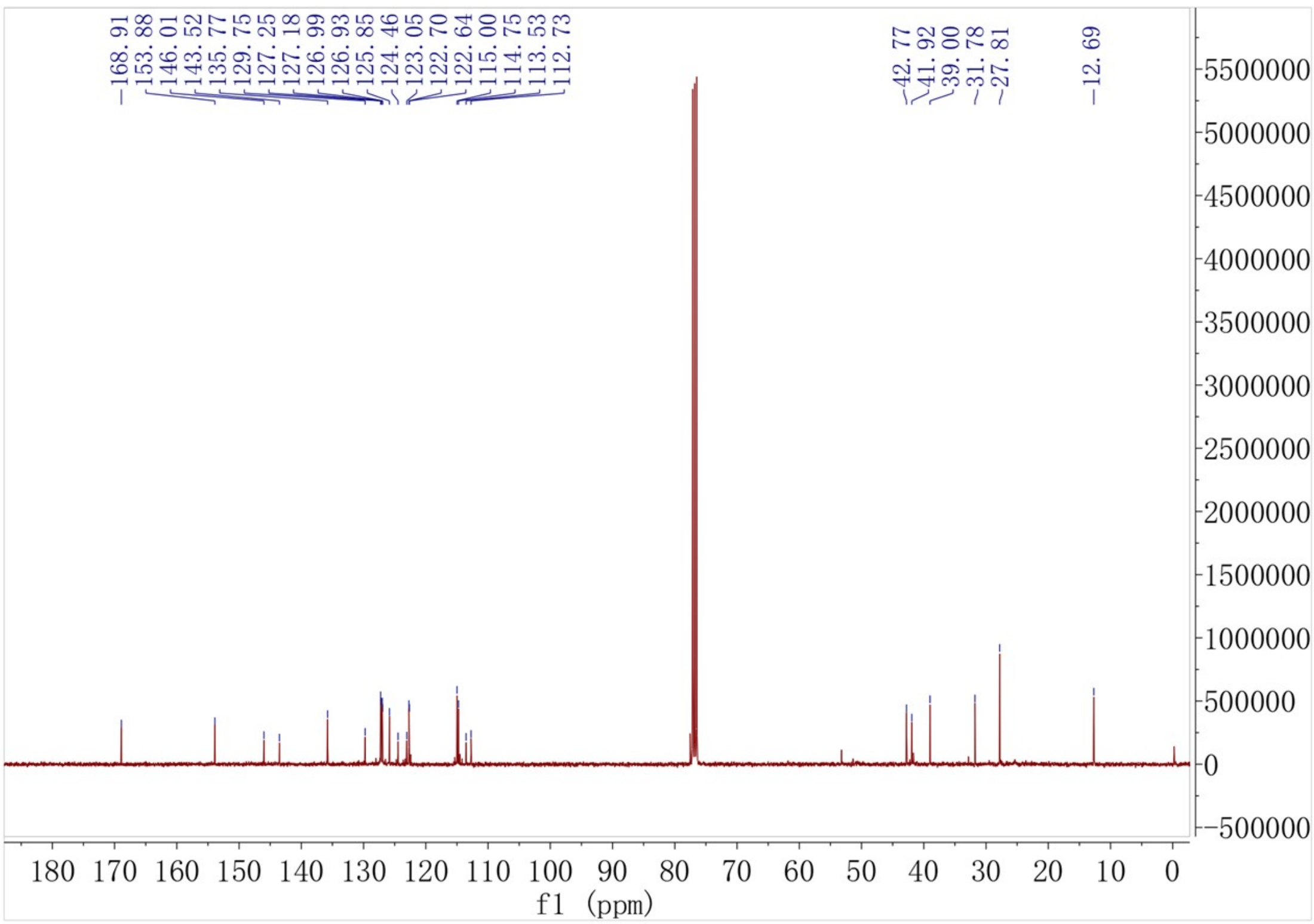

Fig. S12 ^{13}C NMR of **DPTZ** in $CDCl_3$

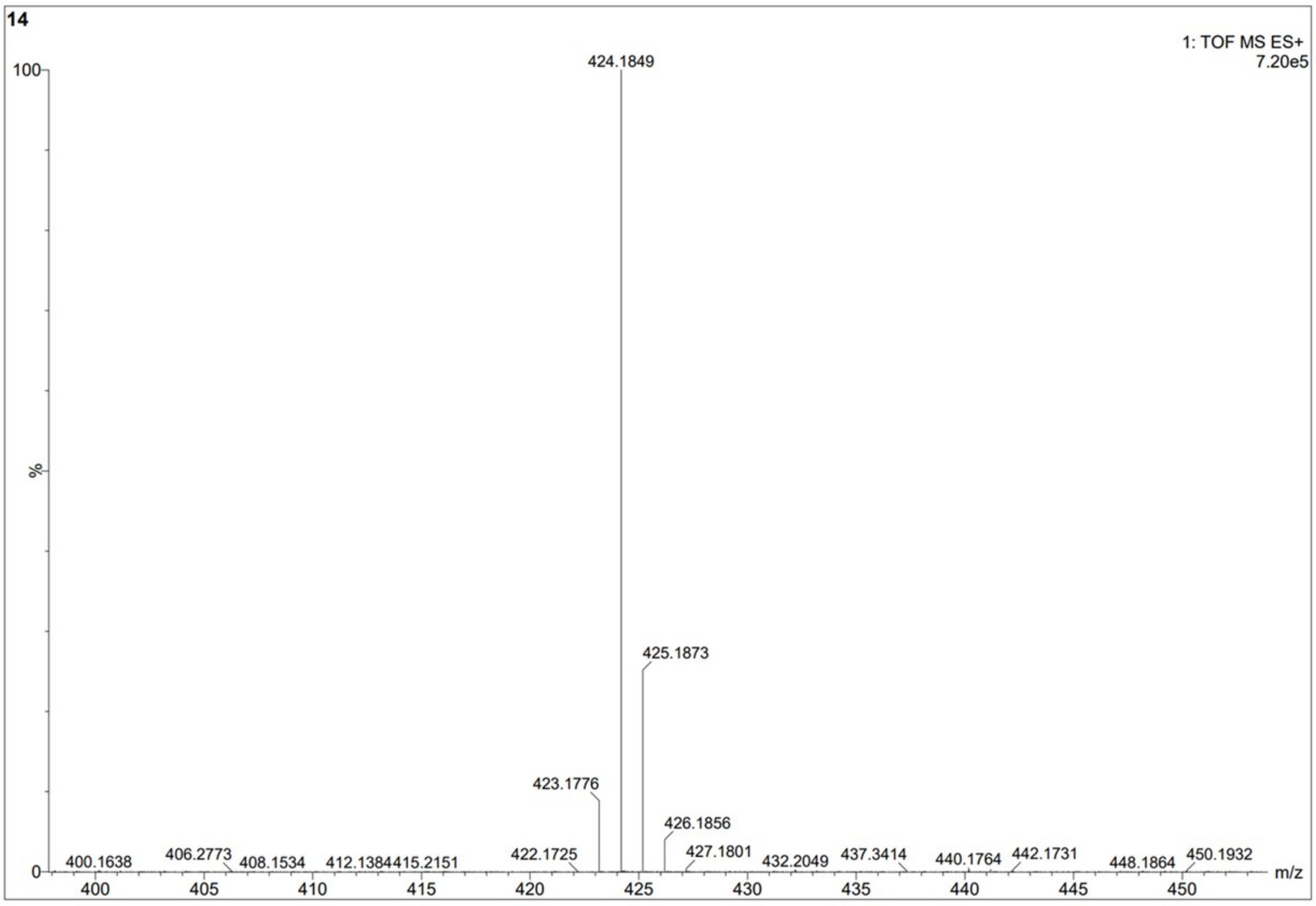

Fig. S13 HRMS of **DPTZ**.

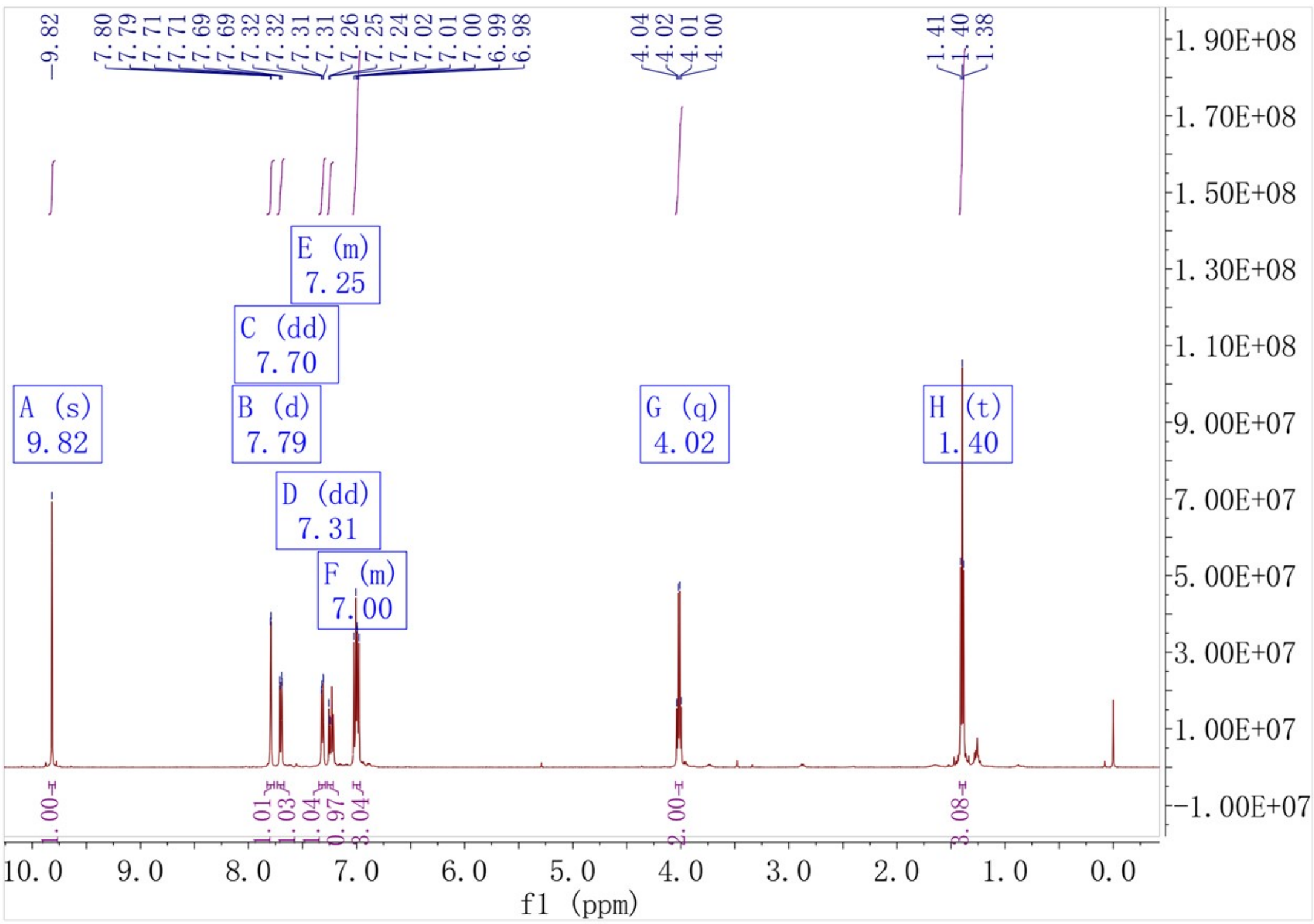

Fig. S14 ^{1}H NMR of **PSZ** in $CDCl_3$

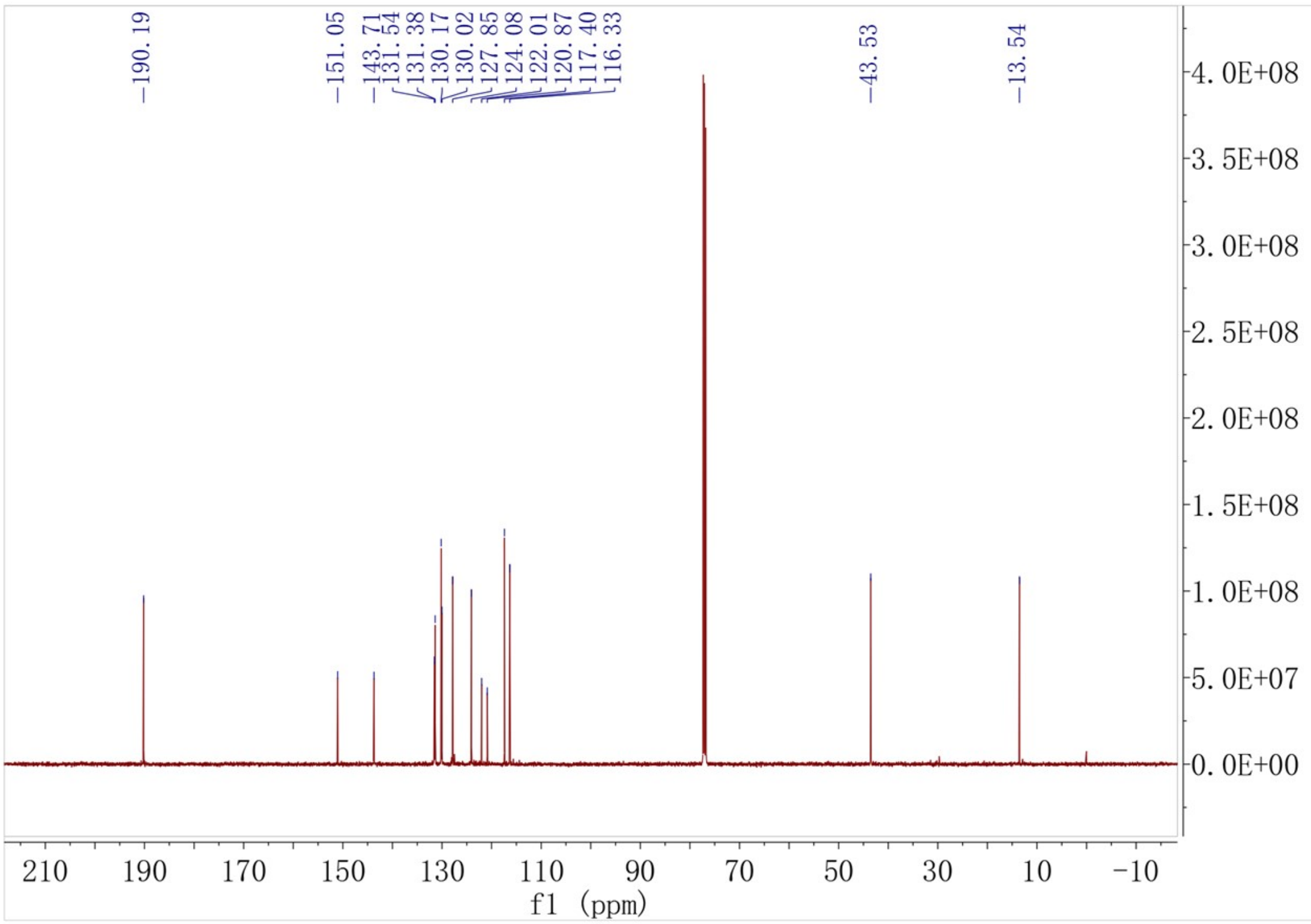

Fig. S15 ^{13}C NMR of **PSZ** in $CDCl_3$

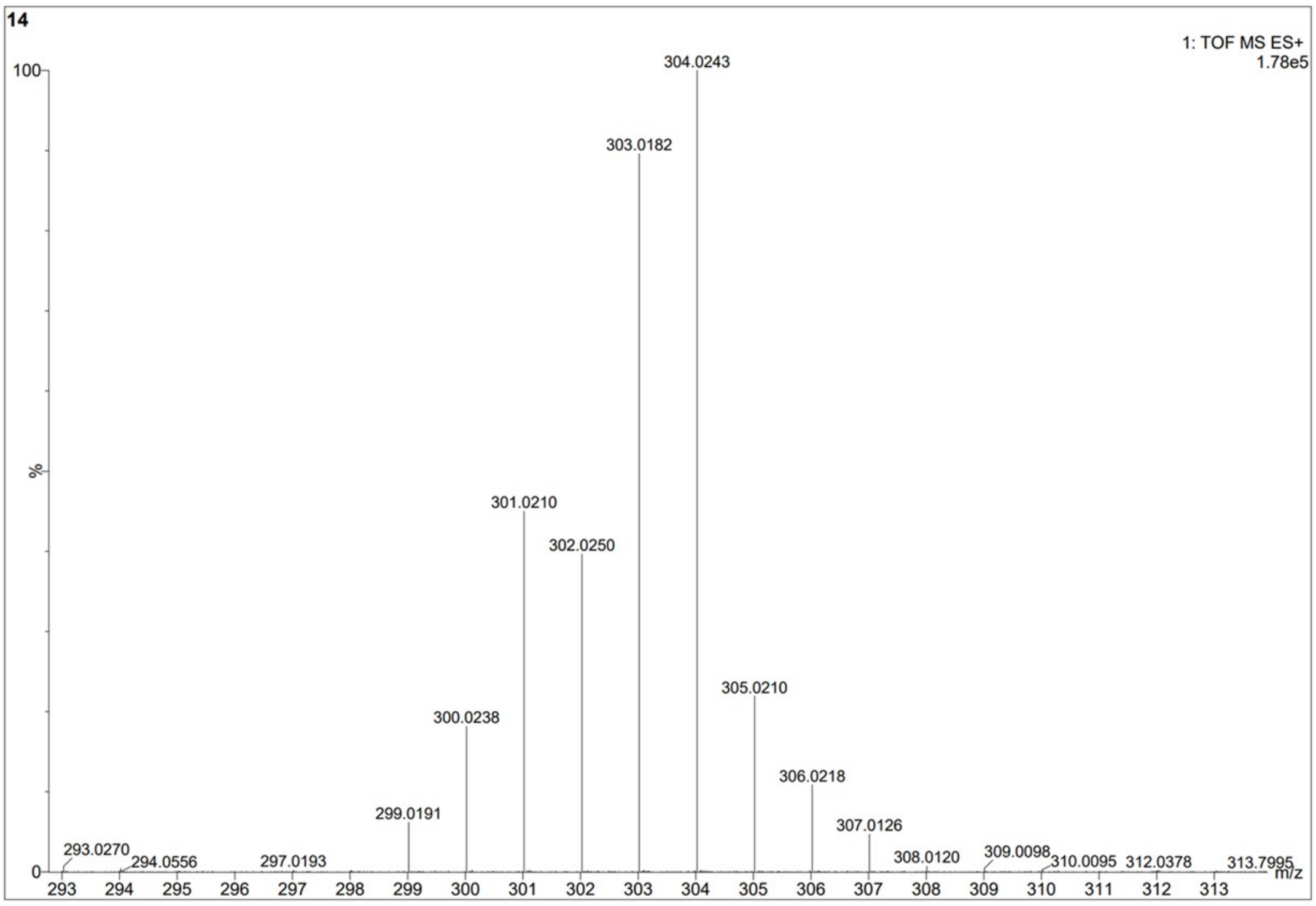

Fig. S16 HRMS of **PSZ**.

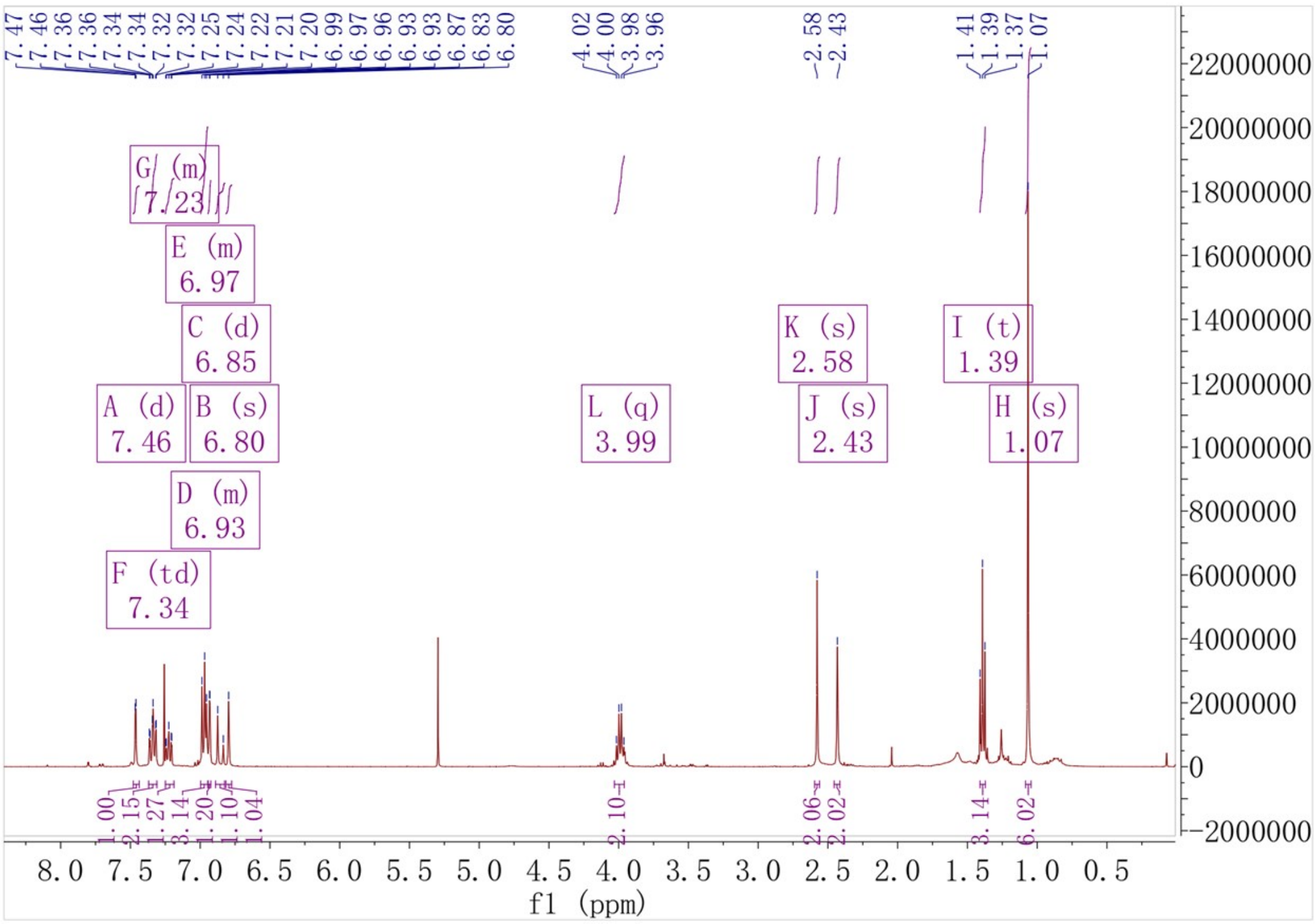

Fig. S17 ^{1}H NMR of **DPSZ** in $CDCl_3$

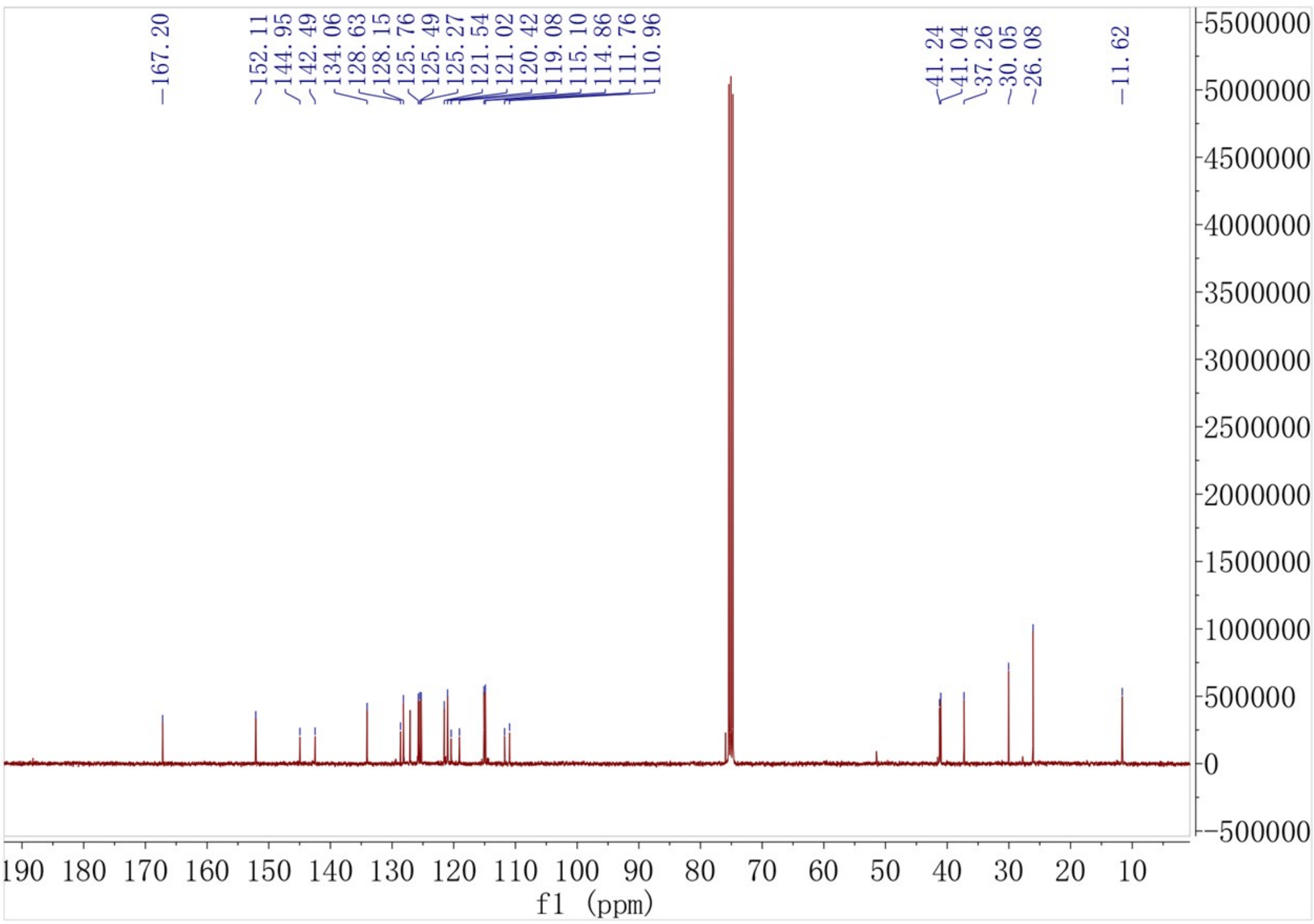

Fig. S18 ^{13}C NMR of **DPSZ** in $CDCl_3$

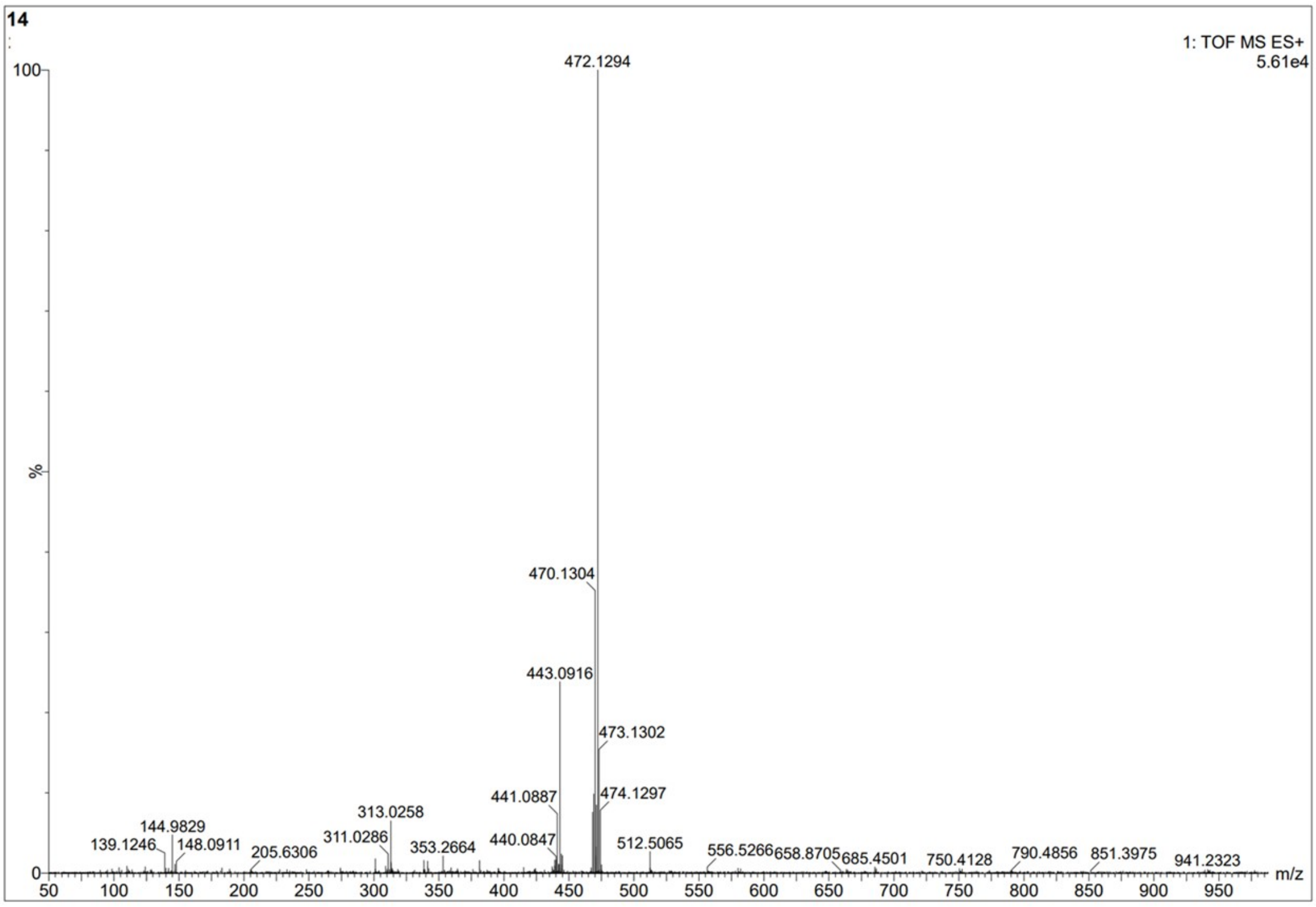

Fig. S19 HRMS of **DPSZ**.
